# The French Integrative Psychosocial Rehabilitation Assessment for Complex Situations (FIPRACS): Modelization of an Adapted Assessment Method Toward Long-Term Psychiatric Inpatients With Disabling, Severe and Persistent Mental Illness

**DOI:** 10.3389/fpsyt.2020.540680

**Published:** 2020-09-18

**Authors:** Christophe Clesse, S. Salime, I. Dumand, S. Barbier Concetta-Ciciarelli, S. Lavenir, K. Kacemi, P. Heckel-Chalet, Frank Sissung, Aurore Poinsignon, Anthony Simon, M. Decker, M. Batt

**Affiliations:** ^1^ Center for Psychiatry, Wolfson Institute of Preventive Medicine, Barth & The London School of Medicine & Dentistry, Queen Mary University of London, London, United Kingdom; ^2^ Centre Hospitalier de Jury-les-Metz, Metz, France; ^3^ Laboratoire INTERPSY (EA 4432), Université de Lorraine, Nancy, France; ^4^ IREPS Grand-Est, Laxou, France; ^5^ Association Espoir 54, Nancy, France; ^6^ Association d’Information et d’Entraide Mosellane, Metz, France; ^7^ Association Famille Rurale de Moselle, Solgne, France

**Keywords:** psychosocial rehabilitation (PSR), psychological assessment, Rorschach, anamnesis, chronic psychosis, neuropsychological evaluation, rehab, integrative assessment

## Abstract

For the past forty years, the generalization of community-based approaches has prompted psychiatry into promoting a deinstitutionalization movement and a psychosocial rehabilitation approach (PSR) for individuals with schizophrenia and related difficulties. Unfortunately, this approach generally does not involve the most severe cognitive and psycho-affective clinical situations among this population despite an increasing number of publications advocating that all individuals should be included in PSR and deinstitutionalization programs. In this context, considering the absence of an assessment battery designed for French individuals with particularly disabling, severe, and persistent mental illness (IDSPMI), we constructed an integrative assessment model adapted to this specific population. To select the most suitable tools for this population, a literature review (inspired by the PRISMA protocol) and a systematic review were combined with a clinical assessment study. The literature review first identified the cognitive and psycho-affective functions which mainly influence the day-to-day life adaptation of individuals engaged in a PSR/deinstitutionalization program. The systematic review then gathered all of the useable French validated tools to assess the initially selected dimensions (n = 87). To finish, for each dimension, the selected 87 tools were included in a clinical assessment study performed within a French psychiatric hospital. The authors collected and verified the characteristics of each tool (validity, French norms, French version, the average speed of the test, ease of use, ability to assess other dimensions). Their suitability was also assessed when applied to IDSPMI. Based on this final clinical evaluation, the authors selected one tool per function to create the French Integrative Psychosocial Rehabilitation Assessment for Complex Situations (FIPRACS). This battery is an assessment tailored to the neurocognitive and psycho-affective potentials of IDSPMI. While further validation studies of this battery are ultimately required, the practical/clinical implications of this battery are presented and discussed.

## Introduction

For nearly 40 years, Psychosocial Rehabilitation (PSR) which is «a process that facilitates the opportunity for individuals [ … ] to reach their optimal level of independent functioning in the community» (1) has profoundly shaped the mental health system of many Western countries. In a biopsychosocial conceptualization model, PSR can combine deinstitutionalization programs (2), mental health supported accommodation services (3), psychiatric rehabilitation accompaniment modelization, and social interventions in the community field (4). The purpose of these conceptualizations is thus to promote a movement of empowerment which induces a recovery process for which the trajectory and its related outcomes are beneficial for the individuals (5). In this context, French psychiatry has also undergone profound conceptual and ideological changes accompanied by a continuous adjustment in laws, care policies, as well as in professional practices (6). Henceforth, discouraged by a better understanding of its iatrogenic effects, psychiatric institutionalization is no longer considered as the main relevant response to chronic mental health disorders. To date, there has been a generalization of deinstitutionalization policies associated with community approaches aimed at maintaining individuals with severe chronic psychiatric symptoms within the community (2, 6, 7).

While this dynamic approach hinges on several factors such as professional/financial resources, lack of healthcare diversity and health team organization (8), deinstitutionalization is also contingent on the subsets of populations involved. Recent studies have indeed shown that compared to the general inpatient population suffering from chronic psychiatric disorders, a small group of individuals with disabling, severe and persistent mental illness (IDSPMI) are often unable to readily find their place within the deinstitutionalization movement (9). Population studies reveal that these individuals are generally men, aged between 30 and 60 years (10), with a long psychiatric history (11), predominantly suffering from schizophrenia and/or mental disability (9–11), presenting a lack of autonomy (9–11) and in whom epidemiological prevalence varies according to health territories (10, 11). Estimated between 0.8 and 2.6% of the French population attending psychiatric hospitals, long-term hospitalized IDSPMI (LTHIDSPMI) account for 20 to 25–30% of the total number of hospital day counts per psychiatric institutions (9–11). Faced with this issue, numerous European and French psychiatric hospitals have developed specific PSR modalities such as mental health support accommodation teams to ensure and stabilize a place in the community for these individuals in spite of their specific clinical and social needs (12, 13). These hospitalization alternatives often include a sanitary and social accompaniment allowing users to be part of the community outside of the hospital walls.

Upstream of the deinstitutionalization process, there then arises the difficult question of which individuals, in the selection and orientation of LTHIDSPMI, would ultimately benefit from such a dynamic approach. Unfortunately, given that this process is still generally designed by healthcare teams according to their clinical perceptions of these patients, many selection biases can occur leading to a loss of opportunity for many patients and notably for those who carry the most severe clinical symptoms. This situation is particularly relevant when inpatients do not clinically present a potential to deinstitutionalization because suffocated by the iatrogenic effects of institutionalization (14). In some institutions, some inpatients can also be pressured by teams to engage in a deinstitutionalization program where the supported accommodation could not be adjusted to their psychoaffective symptoms, their desires/expectations, and their neurocognitive potentials. Finally, certain patients such as the aged-LTHIDSPMI can be victims of stigmatization and excluded from deinstitutionalization programs (15–17).

In light of the above, the use of evaluative tests and tools such as the MATRICS consensus cognitive battery can prevent such situations (18, 19). Unfortunately, despite two French book chapters presenting a neuropsychological assessment for general PSR populations (20, 21), there is currently no assessment battery specifically adapted for the French-speaking IDSPMI. Moreover, in French PSR approaches, there is still no integrative assessment able to encompass and evaluate the influence of the subject’s personality on his or her adaptation capabilities. This is particularly important since these elements significantly influence the clinical management of the teams (22) and the personal adequacy of IDSPMI with the available support housing method (group, intensity of stimulations, *etc.*). As a result, the present authors formalized an integrative (neurocognitive and psychoaffective) and multi-professional assessment method entitled “French Integrative Psychosocial Rehabilitation Assessment for Complex Situations (FIPRACS)’’ specifying each assessed dimension, its functional impact and the most appropriate tools for the evaluation of IDSPMI.

## Methods

In order to build the FIPRACS, the INTERPSY Laboratory (EA 4432), the Jury les Metz Hospital Center (23) and partners of the social (*Association d’Information et d’Entraide Mosellane/AIEM*) and medico-social (*Famille Rurales*) sector have created a work structure governed under the seal of shared secrecy (Decree n° 2016-994 of July 20, 2016). The aim of this group was to combine research and clinical practice to secure and adjust the guidelines within the available housing support encompassing this endeavor. Two separate literature reviews (inspired by the Prisma method) (24) and a clinical study were conducted to build the FIPRACS. The first review aimed to identify the key elements required for the adaptation of an individual particularly when entering in a PSR/deinstitutionalization program. This first step helped the research group to conduct a second systematic review which aimed to gather (according to each dimension selected in the first systematic review) all existing tools validated and useable in French for the psychological assessment in PSR. Thereafter, based on this selection, the team conducted a clinical assessment of the usability of the selected tools to identify those most suitable for the Persons with Severe and Disabling Mental Disorders given that their clinical characteristics necessitate specifically adapted tools.

### First Literature Review: The Required Dimension Involved in Adaptation of Individuals When Entering PSR/Deinstitutionalization Programs

**Figure 1:**
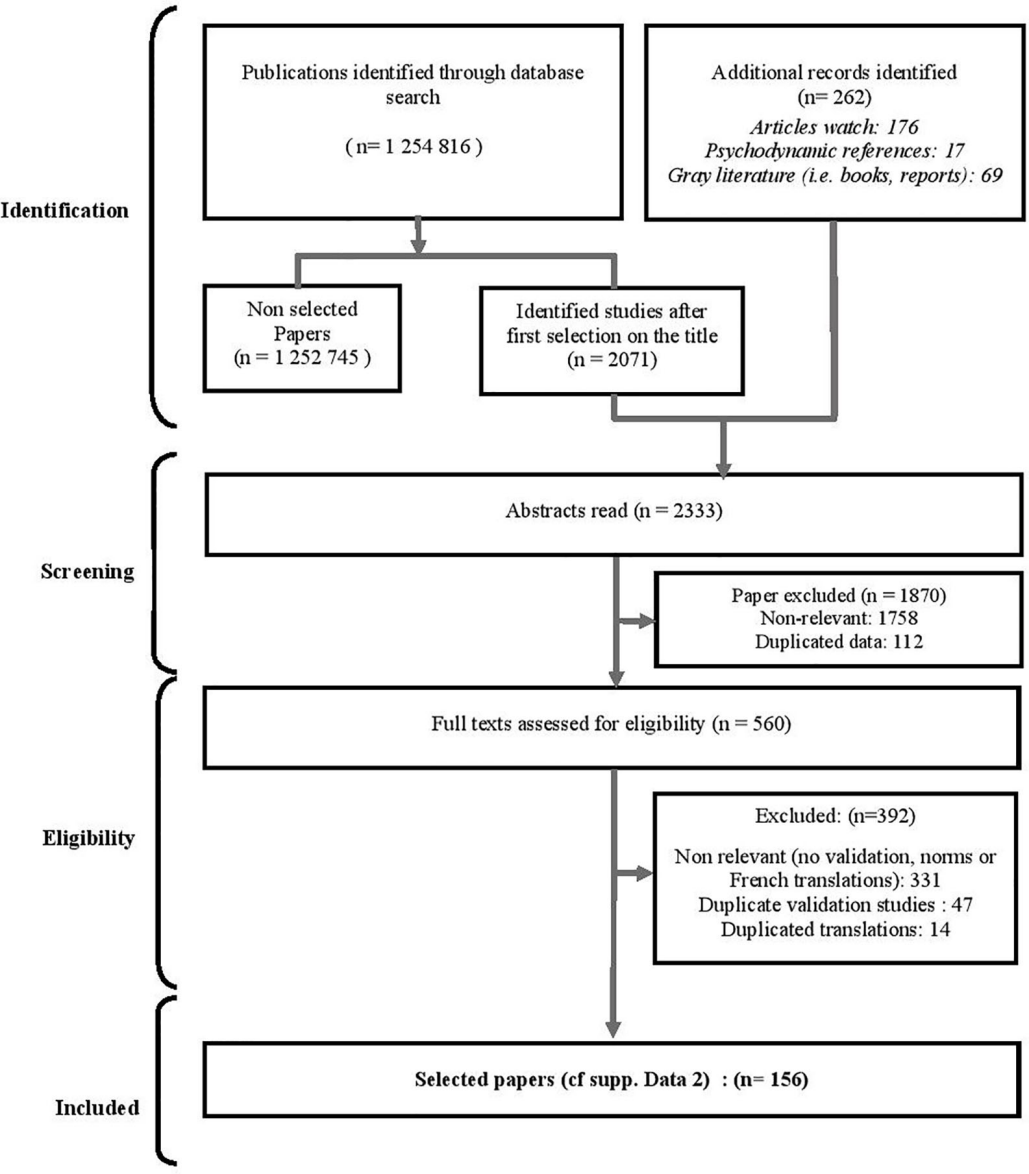
Flow diagram of the selection process on dimensions involved in the adaptation of individuals when entering in a PSR/deinstitutionalization program.

This literature review (**Figure 1**), conducted in both English and French, identified the useful elements for the adaptation of IDSPMI (**Supplemental Data 1**). A double-blind selection process (carried out by the first two authors of this article) was based on the association of two lists of keywords «*‘‘Fonction cognitive’’/’’Cognitive function’’*; *‘‘Cognition’’/’’Cognition’’*; *‘‘Processus mentaux’’/’’Mental process’’*» and «*‘‘Adaptation’’/’’Adaptation’’*; *‘‘Réhabilitation psychosociale’’/’’Psychosocial Rehabilitation’’*; *‘‘Insertion sociale’’/’’Social insertion’’*; *‘‘Bilan psychologique’’/’’Psychological assessment’’*» utilized in nine databases *«Biomed, Cairn, Cochrane, Embase, PsycINFO, PsycARTICLES, PubMed (Medline), ScienceDirect and Web of Science».* For the inclusion criteria, the most complete and representative articles regarding the theoretical models chosen were selected for each dimension. This selection was complemented with recent articles selected for their strong methodological quality and their adequation to our target population. The exclusion criteria were based on the scope of our research, the quality and relevance of scientific information and the comprehensiveness and adequacy with our target population. Finally, when several publications were selected for the same dimension, the latter were restricted to the minimum by relying on the inclusion and exclusion criteria in order to limit the number of selected references.

Among all database results (47,567), a first selection (427 documents) was based on the title of the articles. An additional 262 references were added to this initial selection (176 from a previous article watch carried out by the research team, 69 from the gray literature and 17 psychodynamic texts). After excluding 357 articles based on the abstracts, 335 articles were reviewed of which 296 were ultimately excluded. This process was then updated in December 2019 allowing the addition of 19 articles and the deletion of 23 articles initially selected in 2015. In total, 39 articles were retained describing the following: six neurocognitive functions (*Attention, Processing Speed, Memory, Executive functions, Social cognition and Metacognition*), a set of psychoaffective elements, the clinical and social history of the subject and the psychiatric evaluation. These 39 publications thus provide an up-to-date theoretical insight on the question of the assessment proposed to IDSPMI. In addition, when pertaining to a cognitive function or psychodynamic element, these components present both a current vision of this function/element, a description of the specific impairments experienced by the concerned individuals and an overview on their functional impact. The results of this 1^st^ review are presented in the first part of the *Results* section.

### Second Systematic Review: The Assessment Tools Designed for Individuals With Psychiatric Difficulties

**Figure 2:**
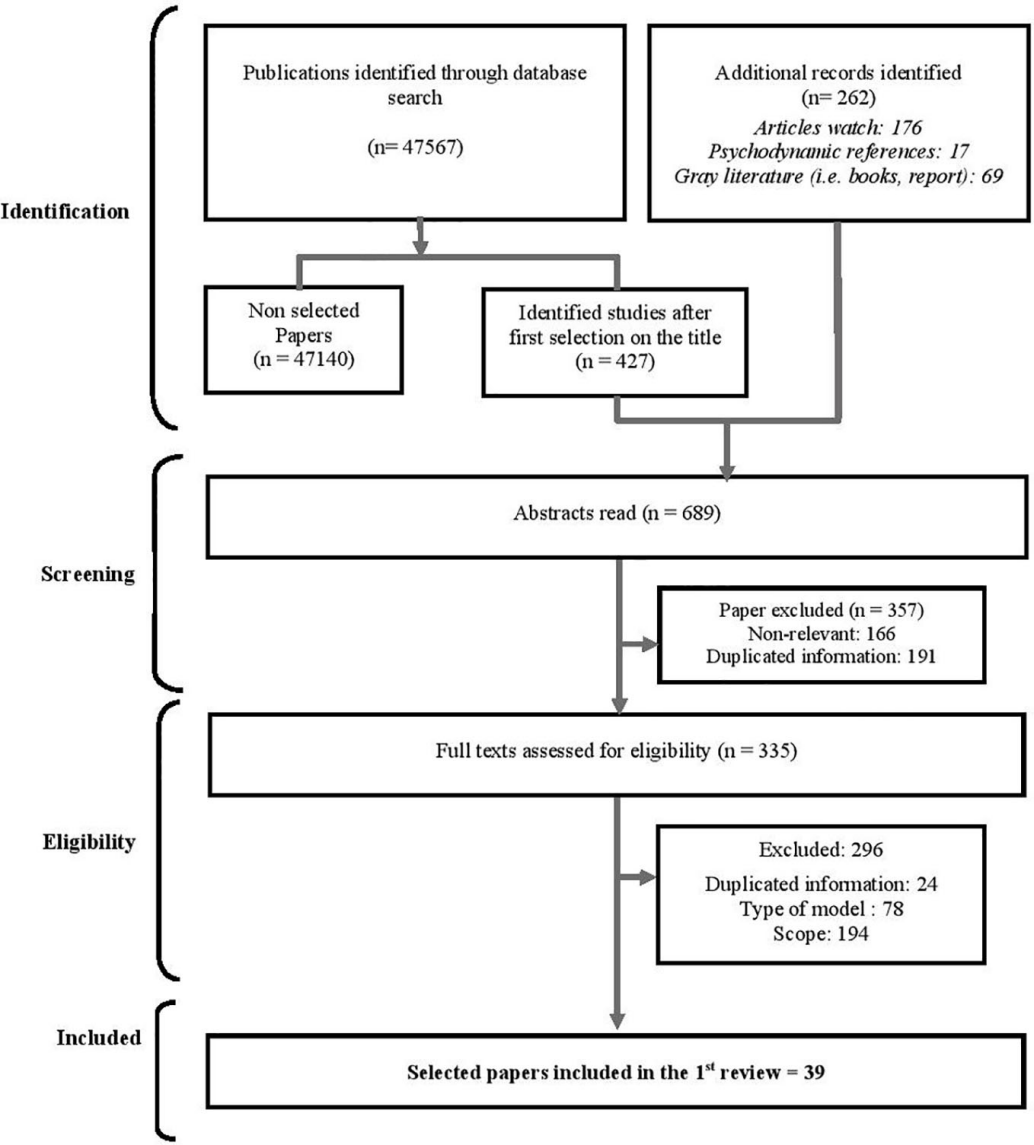
Flow diagram of the selection process on the main selected tools available for neurocognitive evaluation in French language.

In a second step, the first two authors conducted a systematic review of the literature (**Figure 2**) in English and French aimed at identifying the tools allowing the assessment of the psychoaffective aspects and cognitive functions identified by the 1^st^ literature review (**Supplemental Data 1**). Within the nine aforementioned databases, the authors used the combination of a first list of keywords «*‘‘attention’’/’’attention’’*, *‘‘vitesse de traitement’’/’’processing speed’’*, *‘‘mémoire’’/’’memory’’*, *‘‘fonctions exécutives’’/’’executive functions’’*, *‘‘cognition sociale’’/’’social cognition’’*, *‘‘métacognition’’/’’metacognition’’*» with the following second list of keywords: «*‘‘test psychologique’’/’’psychological test’’*, *‘‘test’’/’’test’’, ‘‘outil’’/’’tool’’*, *‘‘évaluation’’/’’evaluation ‘‘*,*’’bilan’’/’’assessment’’, ‘‘validation’’/’’validation’’*».

The inclusion criteria included all publications presenting a useful valid tool to assess each previously identified cognitive dimension: *Attention, Processing Speed, Memory, Executive functions, Social cognition, and Metacognition*. When necessary (*e.g.* due to the verbal support of the test), publications presenting French versions and French norms associated with valid selected tools were included. Exclusion criterion for this selection process was the scope and only four references per tool were selected in order to reduce the number of selected references. The databases revealed 1,254,816 outcomes. The selection conducted based on the title of the articles retained 2,071 articles, to which were incorporated the same 262 additional documents added in the first review process. A total of 2,333 abstracts were screened, leading to the exclusion of 1,870 articles. Of the 560 documents retained and reviewed in their entirety, the authors excluded 301 publications which did not present international or French validation data, or French standards and/or translations. A further 77 articles were excluded in which only one or two validation studies per test were selected by the authors, while 14 articles were rejected since they presented duplicate translation data. This entire process led to a final selection of 156 documents grouping together 87 tools. These tools are classified according to their cognitive function (**Supplemental Data 2, Tables 1–6**). The results of this systematic review are presented in the second part of the *Results* section.

### Assessment Study: Adaptation of the Tools to IDSPMI and Creation of the FICPRACS

Lastly, the interdisciplinary team clinically tested the 87 tools addressing the previously identified six cognitive functions (*Attention, Processing Speed, Memory, Executive functions, Social cognition and Metacognition)* within the PSR teams of the Jury les Metz hospital. Each tool was used with two different patients of the Rehab units of the Jury les Metz hospital for clinical purposes. The spontaneous feedback of the patients as well as test adequation were respectively noted and assessed by the clinician. These observations were reported in a monthly discussion meeting which also aimed to evaluate the speed of the test, the ease-of-use of the tools, the ability to assess other functions and the adaptability of the tools to the clinical specificities of IDSPMI (**Supplemental Data 2, Tables 1–6**). Results for each tool are presented in **Supplemental Data 2**. Authorization to use the clinical data was delivered by the Ethics Committee of the Jury les Metz hospital. Finally, this process enabled to select 14 tools both validated and useable in French (including two complementary test batteries). These 14 tools are considered as the most appropriate for IDSPMI assessment and thus constituting the French Integrative Psychosocial Rehabilitation Assessment for Complex Situations (FIPRACS). This battery is presented in the second part of the *Results* section.

## Results

### Results of the 1^st^ Literature Review: Main Dimensions Involved in the Day-to-Day Life Adaptation of IDSPMI

Relying on 39 selected references selected (**Figure 1**), six neurocognitive dimensions (*attentional capabilities, processing speed, memory skills, executive functions, social cognition, and metacognition*), a set of psychoaffective dimensions and the historical, interpersonal and social background of the IDPMSI were identified as key components of the day-to-day life of ISDPMI involved in a PSR process.

#### Attentional Capacities

Attentional capacities (ACs) allow the subject to process all perceptual information to which he or she is subjected to. They are comprised of three sub-systems described by Posner and Petersen (25). Sustained attention allows the subject to stay focused on a percept or cognitive task for a given time (*ibid.*). Divided attention allows the subject to distribute his attentional functions over several cognitive percepts or tasks at the same time (*ibid.*). Finally, selective attention allows focusing on a percept or a task while inhibiting surrounding percepts considered to be irrelevant (*ibid.*). Nowadays, it is considered that attentional control capacities belong to working memory and that they act in computational synergism with other cognitive functions such as executive functions (26, 27). In individuals with schizophrenia, ACs are considered one of the most impaired cognitive functions (28). This impairment is sometimes increased by treatments, motivation (29), negative or positive psychotic dimensions, and psychoaffective symptomatology (30). With regard to functional impairment, it affects the processing capacities and cognitive functions computationally associated with AC (26). The functional incidence of an AC impairment has a strong impact on the success of a life project driven by a PSR approach.

#### Processing Speed

Defined as the speed with which various cognitive operations are performed, processing speed (PS) is one of the most affected cognitive functions in instances of schizophrenia (28, 31). This impairment is also increased by drug treatments, motivation (29) and psychotic symptoms (30). At the functional level, PS has an influence on encoding, the use of adaptive strategies and decision-making processes. Despite ongoing debate regarding the type of cognitive model and psychometric assessment associated with processing speed (32), it appears that PS primarily interacts with working memory and executive functions (33, 34). Thus, while cognitive remediation enables an improvement in PS, it is acknowledged that the latter is predictive of an improvement in functional abilities (35).

#### Memory Skills

Memory is comprised of five subsystems (procedural memory, perceptive memory, semantic memory, episodic memory (EM) and working memory (WM)) allowing for the processing and maintenance of information (36, 37). Sparsely studied in patients with schizophrenia since they are relatively well-preserved (21), perceptive memory (sensory information), procedural memory (motor tasks) and semantic memory (facts, ideas, concepts, states of meaning and vocabulary) are not particularly considered as main key dimensions in PSR programs.

Conversely, working memory is generally affected in schizophrenic patients and more specifically in IDSPMI (27). Classically considered as a component of executive control, WM is a temporary storage and processing space with limited capacity allowing the performance of complex tasks such as understanding or reasoning (38). It is based on three subsystems (the phonological loop, the visuospatial sketchpad and the episodic buffer ensuring dialogue with the EM) coordinated by a central administrator (*ibid.*). The central executor is responsible for executive attentional control and for the processing and retrieval of information (38, 39). The phonological loop (which is linked to memory span) is used for the temporary storage of acoustic and vocal elements. A saturation of the phonological loop can hence generate interactional difficulties such as hindering the recording of verbal instructions. Finally, the visuospatial sketchpad allows the temporary storage of visual and spatial information (38).

As a part of declarative memory (episodic memory + semantic memory), episodic memory (EM) allows the recovery of previous experiences associated with a spatio-temporal context and an authentic subjective experience involving a capacity for self-apprehension (36, 37). In IDSPMI (31), as in patients with deficit schizophrenia (40), EM is often altered. This alteration is linked to the encoding and retrieval conditions, particularly when the individual is subjected to a high cognitive demand involving control and connectivity functions (41). At the functional level, EM difficulties strongly hamper social and professional adaptation, but also rehabilitation and reeducation work (42). Improvement in EM skills in IDPMSI can be expected with cognitive remediation (35).

#### Executive Functions

Executive functions (EF) are high-level cognitive processes (Inhibition, Cognitive flexibility, WM updating and Planning) involving the fronto-cingulo-parietal network (43). They have an influence on lower cognitive processes and allow individuals to regulate and organize their thoughts and actions according to a behavioral goal (44). Although these four cognitive processes are presented separately, some can act synergistically in the functioning of a more complex process (*e.g.* planning) (*ibid.*). Currently, a deficit in executive functions commonly observed in IDSPMI (28) can lead to organizational and adaptability difficulties impeding the construction of adaptive strategies and self-empowerment. These difficulties must therefore be understood to guide the choice of the working axes in cognitive rehabilitation and training in social abilities considered as beneficial on EF (45).

#### Metacognition

Currently considered to be the central node component of biopsychosocial functioning (46), metacognition is the ability of ‘‘thinking about thinking’’, to have an integrated sense of self through the knowledge and awareness of our cognitive processes (47). Metacognition is divided into two groups of processes: metacognitive knowledge and metacognitive regulation (48). Metacognitive regulation encompasses reflexive awareness (self-awareness), the awareness of others as well as the ability to decenter (47–49). Metacognitive knowledge is knowledge related to the behavior of others, of oneself and of cognitive functioning in itself (*ibid.*). One of the characteristics of all metacognitive processes is that they are described as hierarchical. This functioning hence implies the notion of using lower level metacognitive knowledge to build other more complex knowledge, so-called higher-level metacognitive knowledge (*ibid.*). Finally, it is considered that metacognition appears and evolves within an intersubjective context (47). In practice, metacognition allows access to a knowledge of Self and of others, but above all, to use this knowledge to adapt to contextual and environmental changes (47). In the literature, numerous studies have shown the presence of metacognitive deficits in schizophrenic subjects generating numerous difficulties in their everyday life (*ibid.*). Lastly, it has been shown that an improvement in metacognitive abilities promotes the recovery process (*ibid.*), notably owing to an improvement over time in cognitive and social cognition skills (46).

#### Social Cognition

Social cognition (SC) defines ‘‘the mental operations that underlie social interactions, including perceiving, interpreting and generating responses to the intentions, dispositions, and behaviors of others’’ (50). Four sub-dimensions are commonly associated with SC (51): 1) the perception/management of emotions allows identifying and recognizing emotions, but also to manage and regulate the latter (*ibid.*); 2) social perception which consists of interpreting contextual social information and achieving judgments regarding the behavior of others in order to deduce their social and cultural belonging (*ibid.*); 3) attributional style that echoes the manner in which an individual interprets, explains and understands the negative and positive social elements to which he is subjected (*ibid.*); 4) the theory of mind/ToM which allows to represent the mental states of others by making inferences on the intentions and beliefs of others (*ibid.*). Inferences can be of the first order (representations of the mental states of a third party) or second order (representations of the representations of others). Finally, the differentiation of an emotional and cognitive aspect of ToM allows a relevant association with models integrating the notion of affective and cognitive empathy (52, 53). It is well recognized that individuals with schizophrenia display deficits in SC (54) in terms of emotion perception/processing (particularly in identifying emotions), of social perception and theory of mind (55). These elements lead to numerous functional repercussions that impede social adjustment (52, 55). However, they remain stable according to age (56) and can be improved by an increase in metacognitive abilities (46). Given that IDPMSI are directly impacted by these deficits (potentially increased by institutionalization, its codes, and the lack of social interactions), it is therefore imperative to properly assess these components.

#### Psychoaffective Dimensions

There are several psychoaffective elements associated with the subject’s personality and personal experience that can hamper the success of a psychosocial rehabilitation/housing support program in IDPMSI. Although regularly mentioned by clinicians, there is currently no empirical work studying the impact of psychoaffective components on the success of psychosocial rehabilitation programs. Among others, the ability to engage in a therapeutic alliance, transference and countertransference processes, previous experiences, acting out and ability to accept group interactions (particularly for housing support programs) are known to have a significant influence on PSR projects of IDSPMI (23, 57, 58).

#### Historical, Interpersonal, Clinical and Social Dimensions of the Subject

The last selected dimension refers to the historical, clinical, social, and interpersonal background of the patient. Indeed, a growing body of knowledge currently shows that family support, the presence of past professional experiences, a positive financial situation, a recognition of citizenship/individual rights and a supportive social network are considered as factors facilitating the recovery process (59, 60). Similarly, prior clinical information, existing comorbidities and former treatments also represent an important dimension to be collected (61). It is therefore necessary to integrate these aspects in the assessment of IDSPMI willing to enter in a PSR program.

### Results of the 2^nd^ Systematic Review: Systematic Review of the Available Validated Tools to Assess the Neurocognitive Dimensions of IDSPMI

The systematic review carried out here led us to the selection of 156 publications describing 87 potential French translated (or useable without language use *e.g.* stroop) tools. They allow a standardized assessment of the six neurocognitive dimensions previously presented (attentional capabilities, processing speed, memory skills, executive functions, social cognition, and metacognition). Due to their specificity, the tools potentially used to assess the other two identified dimensions (the psychoaffective as well as the historical, interpersonal, clinical and social dimensions of the subject) have not been explored by this systematic review process. These results are available in supplemental data (**Supplemental Data 2**). They contain bibliographic information on the validity, standards, and the presence of French translation of these tools.

### Results of the Assessment Study: Modelization of the FIPRACS Relying on Clinical Assessment

Relying on the two first research steps of this article, the selected tools for the FIPRACS assessment are based on four criteria reported in the supplemental data (**Supplemental Data 2, Tables 1–6**): speed of the test, easse-of-use of the tools, ability to assess other functions and adaptability of the tools to clinical specificities of IDSPMI. According to these assessments, 10 cognitive evaluations have been selected. In addition to, this clinical assessment, four supplemental evaluations devoted to the psychoaffective dimensions and the historical, interpersonal, clinical and social dimensions of the subject were added, yielding a total of 14 tools thus selected to build the FIPRACS. These tools can be grouped into four main fields presented according to their order in the evaluation process: the clinical and social retrospective (anamnesis and life course) analysis, the neurocognitive assessment, the psychoaffective assessment and the psychiatric, clinical and psychosocial assessments.

#### Clinical and Social Retrospective Analysis: Anamnesis, Life Course and Collection of Social Data

The elaboration of the FIPRACS begins with a collection of clinical case history (anamesis) and social (life course evaluation) data referring to the key ‘‘Historical, interpersonal, clinical and social dimensions of the subject’’ dimension. It requires coordination and information sharing between the teams historically involved with the subject, and the current user himself/herself (so as not to dwell on the representations of previous teams involved with the subject). Generally carried out by a paramedical professional (nurse or nursing assistant), the anamnesis is presented as a reconstruction of the medical and social history underlying the reason for follow-up support (61). It encompasses «the chronology of onset of the disorders and the evolutive course of the signs and symptoms» (*ibid.*, p. 5), the presence of personal history (prior hospitalizations, previous diagnoses, addictions, *etc.*) and familial antecedents (proposed diagnoses, symptomatic similarity, *etc.*), the presence of somatic comorbidities, but also the functional consequences of the disorders on the adaptation of the individual (*ibid.*). As part of the FIPRACS, the anamnesis must include a reflection dedicated to the presence of possible cognitive disorders by allocating particular attention to academic and professional history, the presence of learning difficulties or the ability to maintain acquired skills. Lastly, by including the etiology of the disorders within the history of the subject, the anamnesis allows orienting the diagnostic reflection, thereby enabling considering several potential prognoses as well as different care support modalities.

Concomitantly with the anamnesis, the “life course evaluation” focuses on past and present social aspects (previous life projects, social integration, financial data, family ties, places of living, former professions, *etc.*), and allows shedding light on the social adaptation of the subject (62). Associated with an updated collection of social data, the presentation of the “life course” is generally carried out by a social worker or a specialized educator who provides insight on the construction of projects. Finally, it generates self-empowerment when constructed in conjunction with the patient and helps in the selection of the PSR program or the housing support modalities for LTHIDSPMI.

#### The Neuropsychological Evaluation of the FIPRACS

First, among the tools proposed by the literature for attention assessment (**Supplemental Data 2, Table 1**), we retained the *Test of Everyday Attention (TEA)* (63). Despite its length, TEA is the most complete and adjusted tool for IDSPMI. It also allows complementing other function evaluations.

Second, for the assessment of processing speed, these are mainly based on tools aimed at performing a certain number of cognitive tasks during a given time frame (**Supplemental Data 2, Table 2**). For the FIPRACS, we selected the *Stroop test* (64) for its speed, adequation and ease of use with IDSPMI (33). In addition, the conducting of the Stroop test allows assessing other cognitive elements such as inhibition (enabling to reduce the access time of IDSPMI). We also strongly recommend that the examiner assess the delays associated with the use of strategies (caution, verification, inhibition, *etc.*) on PS (65).

Third, the memory skills assessment principally focuses on working memory and episodic memory tools (**Supplemental Data 2, Table 3**). In WM, the central executor is not evaluated since it is studied *via* the attention, procedural speed, long-term memory and executive functions assessment. The selected tool to assess phonological loop is the WAIS IV *‘Backward digit recall’* subtest (66) for its simplicity, ease of use and adequation with IDSPMI. Particular attention must be given to omissions, intrusions, inversions and to any differences greater than two elements between the forward and backward memory span tasks. The chosen assessment tool for the visuospatial sketchpad is the *Rey-Osterrieth Complex Figure Test* (67, 68). This tool has been selected for its ease of execution, speed and capacity to assess other cognitive elements. Moreover, our clinical investigations evaluated its utilization as particularly adapted to IDSPMI. However, it is necessary to combine the *Rey-Osterrieth Complex Figure Test* with a clinical evaluation of the possible influence of other cognitive functions such as executive functions, but also of the influence of psychotic symptoms (69). Lastly, the tool for accessing episodic memory is the *RL-RI 16* test (70). Its ability to dissociate encoding, storage and retrieval processes provides a strong asset for the memory evaluation of IDSPMI. We also recommend being alert to intrusions, perseverance, repetitions and strategies facilitating memorization (*ibid.*). Finally, since *RL-RI* 16 does not capture autobiographical memory which is often deficient in IDSPMI (71), it is useful to associate clinical questioning on this topic.

Fourth, in terms of tools selected for executive functions assessment (**Supplemental Data 2, Table 4**), the *Stroop test* previously used to assess PS (**Supplemental Data 2, Tables 1 and 2**) already allows studying inhibition and attentional control (64). We did not retain any assessment pertaining to WM update considering it is measured by the tools assessing EM and WM. Cognitive flexibility is conversely preferentially studied through the *Wisconsin Card Sorting Test (WCST)* developed by Grant & Berg in 1948 (72). It is notably important to determine the rate of persevering responses and the number of rankings (73). The ability of the *WCST* to assess other cognitive processes in IDSPMI (*ibid.*) and its playful aspect (particularly appealing to our target population) were determining factors in our selection. To assess planning abilities, we retained the *Behavioral Assessment of the Dysexecutive Syndrome (BADS)* battery (74, 75). While this battery presents a reduced speed of use compared to other tools assessing executive function, we noted its ability to assess other functions and its particular adequation with the clinical particularities of IDSPMI. This ecological test thus allows a complete and closer assessment of day-to-day life issues (*ibid.*).

Fifth, among the tools available to assess social cognition (**Supplemental Data, Table 5**) the *Facial Emotions Recognition Test (TREF)* was selected to evaluate the processing of emotions in the FIPRACS (76). This decision was taken given the accuracy of the test, its ease of use, and the recency of its pictures largely appreciated by the IDSPMI. Likewise, we selected *Theory Of Mind-15 (TOM-15)* proposed by Desgranges et al. in 2012 (77) and the French version of the *Interpersonal Reactivity Index (F-IRI)* (78) to assess ToM and empathy. In instances where clinical observation is insufficient, social perception abilities can then be assessed with the *Mini Profile of Nonverbal Sensitivity (MiniPons)* (79). On the other hand, we did not retain a specific tool for attributional style considering that the available tools do not provide additional elements to that of clinical observation. There are also several batteries in French for social cognition (*BICS, ClaCoS, EVACO, PECS-B*) (20). These were not selected for the FIPRACS because their use was associated with a loss of focus and motivation for IDSPMI. Nonetheless, the fact that *EVACO* integrates an assessment of mental handicap as well as a tool pertaining to the capacity to accept assistance (80) leads us to mention the latter as the most suited battery for our target population.

Finally, for metacognition assessment (**Supplemental Data 2, Table 6**), we estimated that the existing questionnaires do not provide additional information to those obtained with clinical interviews and observations. We nonetheless consider that, in addition to clinical evaluation and clinical assessment of insight, the addition of two instructions to the conducting of the *WCST* proposed by Koren et al. (81) allows a quick and relevant estimation of metacognitive abilities. These instructions are: «Between 0 and 100, at what number do you estimate your success in this test?» and «Do you want your answer to count towards the total score». This complementary assessment was integrated in the FIPRACS for its ease of use.

#### Psychoaffective Assessment With the Rorschach Inkblot Test

Within the FIPRACS, the evaluation of psychoaffective elements is based on the Rorschach projective test. This choice is firstly based on the notion that combining psychodynamic and neurocognitive assessments can facilitates the comprehension of clinical and institutional difficulties (82, 83). Furthermore, the Rorschach Assessment Comprehensive System is considered valid for certain psychoaffective aspects owing to a recent meta-analysis (84). Similarly, certain Rorschach indicators using the R-Optimized method have been associated with certain cognitive and social impairments in schizophrenic subjects (85). The model used in the present assessment (psychodynamic model of the Paris school), meanwhile, is currently being validated and features French adult norms (86). The choice of this tool and method was based on the adequacy of the non-figurative material with IDSPMI, its comprehensiveness and the adequacy of the clinical assessments of the subjects observed during our monthly exchanges. The procedure for conducting the Paris school model is based on the enunciation of a non-inductive instruction (*ex: What could that be? »*) followed by the enunciation of the subject’s responses to the ‘‘choices and rejection test’’ followed by the ‘‘localization of responses’’ phase (86, 87).

The interpretation of the Rorschach test is firstly based on a quantitative analysis supported by the psychogram and its underlying rating: Locations, Determinants, Contents, Qualitative section (88). This analysis is based on the French standards published by Tychey et al. (86). More specifically, the F^%^ *(number of F responses/total number of responses × 100)* and the F^+%^ *(number of F^+^ responses and F^+/−^ responses divided by 2, divided by the total of F × 100)* assess the link to concrete reality or conversely to the invasion of the imaginary (86–88). These two indicators allow pinpointing the anchoring and adaptation ability to be anchored in real life of a subject (*ibid*.). Thereafter, a high A^%^ *(number of animal-type responses (A and Ad)/total number of responses × 100)* and a high number of trivial responses (*Ban*) are considered as a sign of preserved socialization abilities, of social conformism and a social adaptability useful for therapeutic commitment (*ibid.*). The AI^%^ *(number of Hd + sex + blood responses × 100/number of responses)*, when greater than 12%, allows determining the presence of disabling anxiety for the subject (*ibid.*). Other indicators such as *the variety of contents* or *the variety of Locations* attest to the flexibility of psychic functioning (*ibid.*) whereas the presence of kinesthetics (movement, posture, relationship) is synonymous with relational capacities (86, 89). Lastly, the number of *“shock”* responses (*Choc R, Choc N, Choc M, Choc au blanc, Eq choc)* allows pinpointing putative traumatic traces or deep psychological impairments depending on the latent content of the card (86, 88).

In terms of qualitative analysis, the use of a self-representation grid (89, 90) attests to the narcissistic foundations and the integrity of the subject’s body image (86). Thereafter, the identification of the object relation modalities of the subject provides insight on the relational valences and potential psychic conflicts (depending on the latent content of the card) that the subject can unfold (91). The ensuing study of the type of anxiety and the identification of the defense mechanisms allow enlightening the team on the subject’s psychoaffective strategies (88). In addition, the sequence of responses *(progredient or regredient processes)* allows ascertaining the presence of an instability or decompensation of the subject and his/her restoration capacities in the event of psychoaffective difficulties (*ibid.*).

The final assessment axis encompasses the subject’s pulsionality and its transformation (92). Based on the vibrancy of the imaginary *(number of responses, number of kinesthetics, F^%^ and F^+%^, the variety of gaps and contents, etc.)*, on mentalization capabilities *(successful affect-representation associations obtained by a combination of drive and formal determinants)* and the quality of symbolization *(qualitative analysis of the responses according to phallic, feminine and aggressive symbolism)*, it is possible to propose an estimate of the subject’s mentalization and symbolization capacities (93). These capabilities allow facing new situations or to demonstrate the ability to develop. In addition, this indicator provides additional light on the presence of operative functioning, the possibilities of acting out as well as certain obstacles to therapeutic development. Lastly, the sensitivity to latent content and the representations of parental images (86) shed insight on the subject’s interactions with the team and his/her social environment.

#### Psychiatric Evaluation, Diagnosis, Assessment of Clinical Elements and Collection of Psychosocial Elements

The last axis of the FIPRACS combines a psychiatric evaluation including the clinical data, the diagnostic elements, the possible reflections pertaining to medication incidence as well as an assessment of clinical and psychosocial elements carried out by the care team, partners and sometimes by family members or loved ones. This collection of observations is furthermore enhanced when supplemented by precise elements stemming from the daily life of the subject. This approach also allows ascertaining the functional repercussions originating from the psychoaffective register, but also to estimate the functional repercussions of the neurocognitive disorders (61). It also benefits from supplementation with the Functional Repercussion Scale (FRS) thus allowing adapting PSR support to the difficulties of daily life of the accompanied individuals (94). Upon completion, this collection allows the emergence of exchanges between the assessing team and the subject being evaluated regarding his/her difficulties and the desired areas for improvement.

## Discussion

As presented, the conceptualization scheme of the French Integrative Psychosocial Rehabilitation Assessment for Complex Situations (FIPRACS) devoted to individuals with disabling, severe and persistent mental illness (IDSPMI) was firstly based on two literature searches and secondly on a clinical evaluation. The FIPRACS integrates 14 tools. It explores the medical and personal history *via* the *anamnesis* and the *life course evaluation*. The neurocognitive components are subsequently assessed with the *TEA* (attention capacities), the *Stroop* test (processing speed), the *RL-RI 16*, *Backward Digit Recall* and *Rey-Osterrieth Complex Figure* tests (memory), the *WCST* (executive functions), the *TREF*, *TOM-15*, *IRI* and, if necessary, the *MiniPons* (social cognition) and finally, the new assessment approach incorporated into the *WCST* introduced by Koren et al. (81) for metacognition. The psychodynamic component is evaluated with the *Rorschach test* while the clinical and social elements are explored by the *FRS*.

The FIPRACS presents the advantage of carrying out an evaluation of the subjects’ active skills (obtained through direct observation of the subject) but also of their unexploited potentials (owing to the standardized psychological assessment). This specificity allows greater inclusion of certain populations (*e.g.* LTHIDSPMI) who have been influenced by the iatrogenic consequences of institutionalization. Indeed, given that institutionalized subjects tend to present adaptive processes that are highly inferior to their actual abilities, the practitioners in the field inherently influenced by their specific professional representations may thus underestimate the competencies of certain individuals or subsets of the population (15–17). The completion of the FIPRACS can hence represent a tool allowing better equity in psychosocial rehabilitation support. It is also possible that the use of the FIPRACS may also facilitate clinical dialogue and the creation of interdisciplinary workspaces enabling the enhancement of professional skills at the service of the user.

In addition, one of the advantages of the FIPRACS is to provide a scaffold for the professionals in the field by enabling them to understand the cognitive and psychoaffective difficulties of the individual being followed. In practice, this allows, on the one hand, to better carry out deinstitutionalization decisions and orientations towards housing-support programs by selecting those more adapted to the clinical (cognitive and psychoaffective) and social difficulties of IDPSMI. The FIPRACS thus allows refining team reflections and the sustainability of partnership agreements by proposing more adequate orientations. Moreover, it allows providing an accurate insight to the partners of psychiatry upstream of the orientation within housing support programs. Finally, it allows a modulation of the follow-up support as a function of the functional impact resulting from the conjunction of the cognitive and psychoaffective difficulties of the subject.

However, although this article presents the theoretical foundations that led to the construction of the FIPRACS battery, this assessment tool needs to be validated before being generalized among French-speaking teams who are working with IDPSMI. At the formal level, the conceptualization of the FIPRACS adapted to IDSPMI is fairly similar to the MATRICS battery (18). One of the two main differences with the latter is that the FIPRACS is specifically adapted to the clinical specificities of French IDPSMI. It is also based on an interdisciplinary and multi-theoretical evaluation enabling to combine the strengths of each theoretical movement and discipline in order to obtain a more adjusted assessment (95, 96). The multi-theoretical approach to the FIPRACS and its actual use in our PSR teams also allowed highlighting that certain cognitive and psycho-emotional disorders were strongly correlated. The hypothesis aimed at examining the simultaneous existence of a cognitive and psychoaffective expression of certain deficits in these IDPSMI could then be a fruitful source of research hypotheses involving the use of the FIPRACS.

### Strengths and Limitations

Based on a dual review of the bilingual literature inspired by the PRISMA protocol along with a clinical assessment, this article is the first to describe the first step leading to the construction of a battery tailored to the clinical specificities of French-speaking IDPSMI. This article also presents an updated collection of knowledge pertaining to the various functions evaluated upstream of a psychosocial rehabilitation process. Moreover, it provides the reader (*via* additional data) with a comprehensive collection of the tools available in French for the assessment of IDSPMI. Finally, this document is the culmination of a collaborative partnership between research and clinical practice and, as such, concentrates a body of theoretical knowledge on psychosocial rehabilitation assessment useful to all practitioners involved in PSR teams as well as to researchers wishing to rely on a standardized approach. One of the limitations of this endeavor is that the target population in the current document presents a certain heterogeneity. Then, even if all 87 clinically evaluated tools are tools validated in French, we acknowledge that our selection was performed on a limited set of 4 elements. Likewise, the FIPRACS does not incorporate certain additional assessments such as those of an occupational therapist.

## Conclusion

Both beneficial to the inclusion of subjects in a recovery process as well as to the psychosocial rehabilitation support team, the FIPRACS fosters the exploration of prospectively mobilizable dimensions, not directly accessible by direct clinical evaluation, by emphasizing the subject’s true potential. Its use promotes an objective understanding of the subject while allowing a relevant enlightenment of the personal context of the individual. The FIPRACS hence allows practitioners to rely on an equitable and objective tool to promote the recovery process of the individual.

## Data Availability Statement

All datasets generated for this study are included in the article/supplementary material.

## Author Contributions

The first author (CC) led the project. He performed the two systematic reviews with the second author (SS). The discrepancies were assessed and evaluated by the Chief Investigator (MB). The first author (CC) and third author (ID) conceptualized the clinical study. The evaluation of the Rorschach assessment has been made by the first author (CC). From the third to the before the penultimate author, all have performed the evaluation, feedback, clinical assessment and reviewed the manuscript according to their specialty (nurses, social workers, psychiatrist). All have been involved in the writing process and the first author has managed the research project while all the other authors contributed by providing feedback.

## Funding

The funder or the projet is Université de Lorraine through the CPER-Arianne project ACETVIE.

## Conflict of Interest

The authors declare that the research was conducted in the absence of any commercial or financial relationships that could be construed as a potential conflict of interest.
