# Supplementary material for: The French Integrative Psychosocial Rehabilitation Assessment for Complex Situations (FIPRACS): Modelization of an Adapted Assessment Method Toward Long-Term Psychiatric Inpatients With Disabling, Severe and Persistent Mental Illness
Source: Front Psychiatry. 2020 Sep 18;11:540680. doi: 10.3389/fpsyt.2020.540680 (PMC7531021; doi:10.3389/fpsyt.2020.540680)
Supplement: Supplementary file 1 [file DataSheet_1.docx]

**BIOMED**

**1st narrative systematic review:** First Outcomes

**Keyword search:** Integral text.

**Exclusion:** /

|  | Bilan Psychologique | Psychological Assessment |
| --- | --- | --- |
| Cognitive function | 1 | 6384 |
| Fonction cognitive | 2 | 6 |
| Cognition | 0 | 18 |
| Processus mentaux | 2 | 2428 |
| Mental processes | 0 | 7633 |
| Adaptation | 9 | 2953 |
| Psychosocial rehabilitation | 0 | 1582 |
| Réhabilitation psychosociale | 0 | 0 |
| Social insertion | 2 | 301 |
| Insertion sociale | 1 | 2 |

**Number of Articles screened: 21134**

**Number of Articles selected on the title: 169**

**2^nd^ narrative systematic review:** First Outcomes

**Keyword search:** Integral text. *****: refined in the title when exceeding 10000 titles on the integral text research.

**Exclusion:** /

|  | Test Psychologique | Psychological test | Test | Outil | Tool | Évaluation | Evaluation | Bilan | Assessment | Validation |
| --- | --- | --- | --- | --- | --- | --- | --- | --- | --- | --- |
| Attention | 17 | 9099 | 4625* | 20 | 368* | 65 | 1342* | 31 | 1114* | 9208 |
| Vitesse de traitement | 11 | 3 | 38 | 11 | 8 | 11 | 29 | 23 | 13 | 7 |
| Processing Speed | 2 | 1694 | 1235* | 4 | 329* | 12 | 9262 | 2 | 8990 | 4676 |
| Mémoire | 3 | 13 | 59 | 3 | 35 | 6 | 47 | 2 | 37 | 19 |
| Memory | 4 | 3527 | 1270* | 3 | 9383 | 16 | 9366 | 5 | 361* | 4219 |
| Fonctions exécutives | 0 | 0 | 0 | 0 | 3 | 0 | 0 | 0 | 0 | 0 |
| Executive functions | 0 | 1421 | 5658 | 1 | 2573 | 11 | 3030 | 1 | 3814 | 917 |
| Cognition sociale | 0 | 12 | 25 | 0 | 9 | 0 | 17 | 0 | 22 | 5 |
| Social cognition | 1 | 2283 | 5719 | 0 | 2475 | 9 | 2950 | 0 | 3937 | 956 |
| Métacognition | 0 | 0 | 0 | 0 | 0 | 0 | 0 | 0 | 0 | 0 |
| Metacognition | 0 | 63 | 141 | 0 | 71 | 1 | 81 | 0 | 109 | 27 |

**Number of Articles screened: 116969**

**Number of Articles selected on the title: 284**

**COCHRANE**

**1st narrative systematic review:** First Outcomes

**Keyword search:** Title, Abstract, Keyword (word variations included). **Exclusion:** /

|  | Bilan Psychologique | Psychological Assessment |
| --- | --- | --- |
| Cognitive function | 0 | 114 |
| Fonction cognitive | 1 | 39 |
| Cognition | 3 | 237 |
| Processus mentaux | 0 | 7 |
| Mental processes | 0 | 42 |
| Adaptation | 1 | 43 |
| Psychosocial rehabilitation | 0 | 19 |
| Réhabilitation psychosociale | 0 | 7 |
| Social insertion | 0 | 4 |
| Insertion sociale | 0 | 4 |

**Number of Articles screened: 521**

**Number of Articles selected on the title: 6**

**2^nd^ narrative systematic review:** First Outcomes

**Keyword search:** Title, Abstract, Keyword (word variations included) **Exclusion:** /

|  | Test Psychologique | Psychological test | Test | Outil | Tool | Évaluation | Evaluation | Bilan | Assessment | Validation |
| --- | --- | --- | --- | --- | --- | --- | --- | --- | --- | --- |
| Attention | 7 | 29 | 79 | 3 | 24 | 55 | 161 | 1 | 256 | 34 |
| Vitesse de traitement | 0 | 2 | 23 | 3 | 5 | 6 | 27 | 0 | 44 | 8 |
| Processing Speed | 0 | 0 | 12 | 1 | 2 | 4 | 18 | 0 | 33 | 4 |
| Mémoire | 2 | 5 | 46 | 5 | 14 | 27 | 59 | 2 | 89 | 9 |
| Memory | 2 | 13 | 80 | 5 | 26 | 33 | 92 | 91 | 150 | 18 |
| Fonctions exécutives | 0 | 0 | 9 | 0 | 3 | 1 | 7 | 1 | 14 | 0 |
| Executive functions | 1 | 5 | 17 | 0 | 5 | 7 | 20 | 1 | 29 | 3 |
| Cognition sociale | 12 | 24 | 42 | 6 | 20 | 37 | 96 | 2 | 132 | 12 |
| Social cognition | 12 | 24 | 42 | 6 | 20 | 38 | 96 | 2 | 132 | 12 |
| Métacognition | 0 | 0 | 0 | 0 | 0 | 0 | 0 | 0 | 2 | 0 |
| Metacognition | 0 | 0 | 0 | 0 | 0 | 0 | 0 | 0 | 2 | 0 |

**Number of Articles screened: 2503**

**Number of Articles selected on the title: 17**

**CAIRN**

**1st narrative systematic review:** First Outcomes

**Keyword search:** Integral text. **Exclusion:** Book

|  | Bilan Psychologique | Psychological Assessment |
| --- | --- | --- |
| Cognitive function | 0 | 5 |
| Fonction cognitive | 3 | 1 |
| Cognition | 198 | 188 |
| Processus mentaux | 11 | 6 |
| Mental processes | 1 | 0 |
| Adaptation | 159 | 130 |
| Psychosocial rehabilitation | 0 | 0 |
| Réhabilitation psychosociale | 2 | 1 |
| Social insertion | 0 | 0 |
| Insertion sociale | 16 | 11 |

**Number of Articles screened: 732**

**Number of Articles selected on the title: 28**

**2^nd^ narrative systematic review:** First Outcomes

**Keyword search:** Integral text. *****: refined in the abstract when more than 10000 titles had to be screened.

**Exclusion:** Book

|  | Test Psychologique | Psychological test | Test | Outil | Tool | Évaluation | Evaluation | Bilan | Assessment | Validation |
| --- | --- | --- | --- | --- | --- | --- | --- | --- | --- | --- |
| Attention | 177 | 30 | 60* | 145* | 3493 | 200* | 200* | 28 | 0* | 30* |
| Vitesse de traitement | 4 | 0 | 173 | 102 | 25 | 182 | 182 | 70 | 71 | 73 |
| Processing Speed | 0 | 0 | 61 | 19 | 9 | 54 | 54 | 14 | 28 | 17 |
| Mémoire | 108 | 16 | 38 | 97* | 1788 | 91* | 91* | 21* | 2624 | 26* |
| Memory | 10 | 10 | 2625 | 2162 | 818 | 2864 | 2864 | 1073 | 903 | 1175 |
| Fonctions exécutives | 10 | 3 | 723 | 388 | 61 | 702 | 702 | 317 | 205 | 300 |
| Executive functions | 5 | 0 | 223 | 87 | 39 | 165 | 165 | 35 | 99 | 61 |
| Cognition sociale | 5 | 3 | 425 | 293 | 30 | 417 | 417 | 113 | 101 | 192 |
| Social cognition | 2 | 0 | 553 | 249 | 62 | 453 | 453 | 77 | 166 | 233 |
| Métacognition | 0 | 0 | 435 | 309 | 44 | 396 | 396 | 132 | 122 | 187 |
| Metacognition | 0 | 0 | 435 | 309 | 44 | 396 | 396 | 132 | 122 | 187 |

**Number of Articles screened: 37481**

**Number of Articles selected on the title: 79**

**EMBASE**

**1st narrative systematic review:** First Outcomes

**Keyword search:** Title, Abstract, Author, Keywords. **Exclusion:** /

|  | Bilan Psychologique | Psychological Assessment |
| --- | --- | --- |
| Cognitive function | 0 | 0 |
| Fonction cognitive | 0 | 30 |
| Cognition | 0 | 75 |
| Processus mentaux | 0 | 0 |
| Mental processes | 0 | 0 |
| Adaptation | 0 | 0 |
| Psychosocial rehabilitation | 0 | 1 |
| Réhabilitation psychosociale | 0 | 0 |
| Social insertion | 0 | 0 |
| Insertion sociale |  |  |

**Number of Articles screened: 106**

**Number of Articles selected on the title: 11**

**2^nd^ narrative systematic review:** First Outcomes

**Keyword search:** Title, Abstract, Author, Keywords. *****: refined on the title when > 10000

**Exclusion:** /

|  | Test Psychologique | Psychological test | Test | Outil | Tool | Évaluation | Evaluation | Bilan | Assessment | Validation |
| --- | --- | --- | --- | --- | --- | --- | --- | --- | --- | --- |
| Attention | 0 | 87 | 575* | 2 | 656 | 131* | 1831* | 1 | 3038* | 4954 |
| Vitesse de traitement | 0 | 0 | 0 | 0 | 0 | 0 | 0 | 0 | 0 | 0 |
| Processing Speed | 0 | 5 | 6459 | 0 | 418 | 25 | 25 | 0 | 2623 | 183 |
| Mémoire | 0 | 0 | 28 | 2 | 37 | 30 | 30 | 0 | 48 | 6 |
| Memory | 0 | 93 | 1538* | 1 | 7391 | 2628 | 2628 | 1 | 4052 | 2704 |
| Fonctions exécutives | 0 | 0 | 5 | 0 | 0 | 5 | 5 | 0 | 2 | 3 |
| Executive functions | 0 | 7 | 6654 | 0 | 496 | 1394* | 1394* | 0 | 3207 | 161 |
| Cognition sociale | 0 | 0 | 3 | 0 | 0 | 522 | 522 | 0 | 1 | 1 |
| Social cognition | 0 | 2 | 4373 | 0 | 253 | 2 | 522 | 0 | 1227 | 112 |
| Métacognition | 0 | 0 | 480 | 0 | 81 | 205 | 205 | 0 | 414 | 36 |
| Metacognition | 0 | 0 | 480 | 0 | 81 | 205 | 205 | 0 | 414 | 36 |

**Number of Articles screened: 65945**

**Number of Articles selected on the title: 167**

**PSYCINFO ET PSYCARTICLE**

**1st narrative systematic review:** First Outcomes

**Keyword search:** Title, Abstract+smart-text. *: exclusion of smart-text

**Exclusion:** /

|  | Bilan Psychologique | Psychological Assessment |
| --- | --- | --- |
| Cognitive function | 187 | 8997 |
| Fonction cognitive | 6 | 19 |
| Cognition | 716 | 3650* |
| Processus mentaux | 0 | 3 |
| Mental processes | 6 | 1841 |
| Adaptation | 173 | 2622* |
| Psychosocial rehabilitation | 5 | 444 |
| Réhabilitation psychosociale | 1 | 5 |
| Social insertion | 1 | 19 |
| Insertion sociale | 2 | 2 |

**Number of Articles screened: 18699**

**Number of Articles selected on the title: 121**

**2^nd^ narrative systematic review:** First Outcomes

**Keyword search:** Abstract/ Exact terms ***:** add of smart-text **: Refined on the title when outcomes > 10000

**Exclusion:** /

|  | Test Psychologique | Psychological test | Test | Outil | Tool | Évaluation | Evaluation | Bilan | Assessment | Validation |
| --- | --- | --- | --- | --- | --- | --- | --- | --- | --- | --- |
| Attention | 14 | 2308 | 754** | 58 | 7920 | 111 | 11512 | 7 | 20169 | 2838* |
| Vitesse de traitement | 1 | 1 | 10 | 2 | 4 | 5 | 5 | 130 | 3 | 372* |
| Processing Speed | 1 | 166 | 4622 | 1 | 341 | 5 | 609 | 9* | 1846 | 2259* |
| Mémoire | 6 | 5 | 221 | 18 | 19 | 39 | 74 | 6 | 34 | 42* |
| Memory | 8 | 1956 | 2373** | 25 | 4814 | 76 | 7219 | 7 | 750** | 2442* |
| Fonctions exécutives | 2 | 2 | 25 | 7 | 6 | 15 | 19 | 3 | 17 | 11* |
| Executive functions | 3 | 343 | 9069 | 8 | 828 | 16 | 1375 | 3 | 4292 | 1381* |
| Cognition sociale | 1 | 1 | 11 | 6 | 8 | 4 | 8 | 23* | 4 | 2414* |
| Social cognition | 2 | 269 | 2777 | 6 | 811 | 5 | 1129 | 55* | 1929 | 9024* |
| Métacognition | 3* | 0 | 1 | 1 | 2 | 0 | 2* | 0 | 2 | 0 |
| Metacognition | 3* | 46 | 670 | 1 | 209 | 1 | 267 | 2* | 534 | 414 |

**Number of Articles screened: 114281**

**Number of Articles selected on the title: 420**

**PUBMED (Medline)**

**1st narrative systematic review:** First Outcomes

**Keyword search:** Title+Abstract

**Exclusion:** /

|  | Bilan Psychologique | Psychological Assessment |
| --- | --- | --- |
| Cognitive function | 0 | 13 |
| Fonction cognitive | 0 | 0 |
| Cognition | 0 | 39 |
| Processus mentaux | 0 | 0 |
| Mental processes | 0 | 2 |
| Adaptation | 0 | 32 |
| Psychosocial rehabilitation | 0 | 1 |
| Réhabilitation psychosociale | 0 | 1 |
| Social insertion | 0 | 0 |
| Insertion sociale | 0 | 0 |

**Number of Articles screened: 88**

**Number of Articles selected on the title: 5**

**2^nd^ narrative systematic review:** First Outcomes

**Keyword search:** Title+Abstract *: Refined on the title when outcomes > 10000

**Exclusion:** Book

|  | Test Psychologique | Psychological test | Test | Outil | Tool | Évaluation | Evaluation | Bilan | Assessment | Validation |
| --- | --- | --- | --- | --- | --- | --- | --- | --- | --- | --- |
| Attention | 0 | 51 | 483* | 1 | 9538 | 382* | 382* | 1 | 411* | 3487 |
| Vitesse de traitement | 0 | 0 | 1 | 0 | 0 | 1 | 1 | 0 | 1 | 0 |
| Processing Speed | 0 | 0 | 28 | 0 | 221 | 467 | 467 | 0 | 1470 | 1 |
| Mémoire | 0 | 0 | 23 | 0 | 15 | 18 | 18 | 0 | 30 | 5 |
| Memory | 1 | 0 | 1243 | 0 | 4740 | 8906 | 8906 | 1 | 533* | 1681 |
| Fonctions exécutives | 0 | 69 | 5 | 0 | 2 | 4 | 4 | 0 | 5 | 2 |
| Executive functions | 0 | 6 | 2805 | 0 | 291 | 673 | 673 | 0 | 1769 | 87 |
| Cognition sociale | 0 | 0 | 5 | 0 | 3 | 1 | 1 | 0 | 2 | 0 |
| Social cognition | 0 | 0 | 1061 | 0 | 169 | 325 | 325 | 0 | 702 | 66 |
| Métacognition | 0 | 0 | 344 | 0 | 73 | 148 | 148 | 0 | 286 | 28 |
| Metacognition | 0 | 0 | 344 | 0 | 73 | 148 | 148 | 0 | 286 | 28 |

**Number of Articles screened: 54623**

**Number of Articles selected on the title: 282**

**SCIENCE DIRECT**

**1st narrative systematic review:** First Outcomes

**Keyword search:** Title, Abstract+keyword.

**Exclusion:** /

|  | Bilan Psychologique | Psychological Assessment |
| --- | --- | --- |
| Cognitive function | 3 | 511 |
| Fonction cognitive | 7 | 19 |
| Cognition | 9 | 286 |
| Processus mentaux | 0 | 1 |
| Mental processes | 1 | 183 |
| Adaptation | 9 | 242 |
| Psychosocial rehabilitation | 2 | 51 |
| Réhabilitation psychosociale | 1 | 5 |
| Social insertion | 1 | 8 |
| Insertion sociale | 2 | 6 |

**Number of Articles screened: 1347**

**Number of Articles selected on the title: 19**

**2^nd^ narrative systematic review:** First Outcomes

**Keyword search:** Title, Abstract, Keywords.

**Exclusion:** /

|  | Test Psychologique | Psychological test | Test | Outil | Tool | Évaluation | Evaluation | Bilan | Assessment | Validation |
| --- | --- | --- | --- | --- | --- | --- | --- | --- | --- | --- |
| Attention | 34 | 856 | 32116 | 242 | 10651 | 31299 | 31299 | 184 | 14753 | 6653 |
| Vitesse de traitement | 8 | 4 | 249 | 35 | 25 | 177 | 177 | 66 | 43 | 30 |
| Processing Speed | 3 | 95 | 11396 | 14 | 5820 | 7655 | 7655 | 6 | 1998 | 3314 |
| Mémoire | 20 | 17 | 592 | 104 | 86 | 415 | 415 | 151 | 144 | 71 |
| Memory | 17 | 734 | 33709 | 78 | 4462 | 15735 | 15735 | 53 | 5870 | 2845 |
| Fonctions exécutives | 8 | 7 | 173 | 29 | 29 | 150 | 150 | 46 | 70 | 21 |
| Executive functions | 3 | 176 | 4623 | 25 | 439 | 1970 | 1970 | 15 | 1958 | 309 |
| Cognition sociale | 4 | 2 | 41 | 21 | 24 | 61 | 61 | 13 | 33 | 11 |
| Social cognition | 6 | 149 | 1856 | 38 | 379 | 1132 | 1132 | 14 | 868 | 247 |
| Métacognition | 1 | 33 | 380 | 12 | 99 | 242 | 242 | 4 | 202 | 53 |
| Metacognition | 1 | 33 | 380 | 12 | 99 | 242 | 242 | 4 | 202 | 53 |

**Number of Articles screened: 268909**

**Number of Articles selected on the title: 285**

**WEB OF SCIENCE**

**1st narrative systematic review:** First Outcomes

**Keyword search:** Topic

**Exclusion:** /

|  | Bilan Psychologique | Psychological Assessment |
| --- | --- | --- |
| Cognitive function | 0 | 2104 |
| Fonction cognitive | 0 | 0 |
| Cognition | 0 | 1170 |
| Processus mentaux | 0 | 0 |
| Mental processes | 0 | 853 |
| Adaptation | 0 | 1191 |
| Psychosocial rehabilitation | 0 | 269 |
| Réhabilitation psychosociale | 0 | 1 |
| Social insertion | 0 | 10 |
| Insertion sociale | 0 | 0 |

**Number of Articles screened: 4940**

**Number of Articles selected on the title: 68**

**2^nd^ narrative systematic review:** First Outcomes

**Keyword search:** Topic *: Articles only (filter of WOS)

**Exclusion:** /

|  | Test Psychologique | Psychological test | Test | Outil | Tool | Évaluation | Evaluation | Bilan | Assessment | Validation |
| --- | --- | --- | --- | --- | --- | --- | --- | --- | --- | --- |
| Attention | 0 | 3640 | 95852* | 4 | 27954* | 36174* | 36174* | 3 | 42757* | 10030* |
| Vitesse de traitement | 0 | 0 | 1 | 0 | 0 | 1 | 1 | 0 | 0 | 0 |
| Processing Speed | 1 | 342 | 30527* | 0 | 13684* | 7699* | 7699* | 0 | 7625 | 4388 |
| Mémoire | 0 | 1 | 26 | 1 | 29 | 24 | 24 | 2 | 24 | 8 |
| Memory | 1 | 3137 | 114323* | 1 | 15541* | 19997* | 19997* | 1 | 24801* | 7207 |
| Fonctions exécutives | 0 | 0 | 3 | 0 | 0 | 3 | 3 | 0 | 2 | 3 |
| Executive functions | 0 | 739 | 19064 | 0 | 1882 | 2877 | 2877 | 0 | 8612 | 1012 |
| Cognition sociale | 0 | 0 | 2 | 0 | 1 | 1 | 1 | 0 | 0 | 0 |
| Social cognition | 0 | 692 | 7531 | 0 | 1551 | 2213 | 2213 | 0 | 3290 | 918 |
| Métacognition | 0 | 94 | 1544 | 0 | 472 | 569 | 569 | 0 | 968 | 241 |
| Metacognition | 0 | 94 | 1544 | 0 | 472 | 569 | 569 | 0 | 968 | 241 |

**Number of Articles screened: 594105**

**Number of Articles selected on the title: 537**
