# Supplementary material for: The French Integrative Psychosocial Rehabilitation Assessment for Complex Situations (FIPRACS): Modelization of an Adapted Assessment Method Toward Long-Term Psychiatric Inpatients With Disabling, Severe and Persistent Mental Illness
Source: Front Psychiatry. 2020 Sep 18;11:540680. doi: 10.3389/fpsyt.2020.540680 (PMC7531021; doi:10.3389/fpsyt.2020.540680)
Supplement: Supplementary file 2 [file DataSheet_2.docx]

**List of the main selected tools available in French Language**

**Table 1: Principal tools useable and available in French language for attention assessment**

**
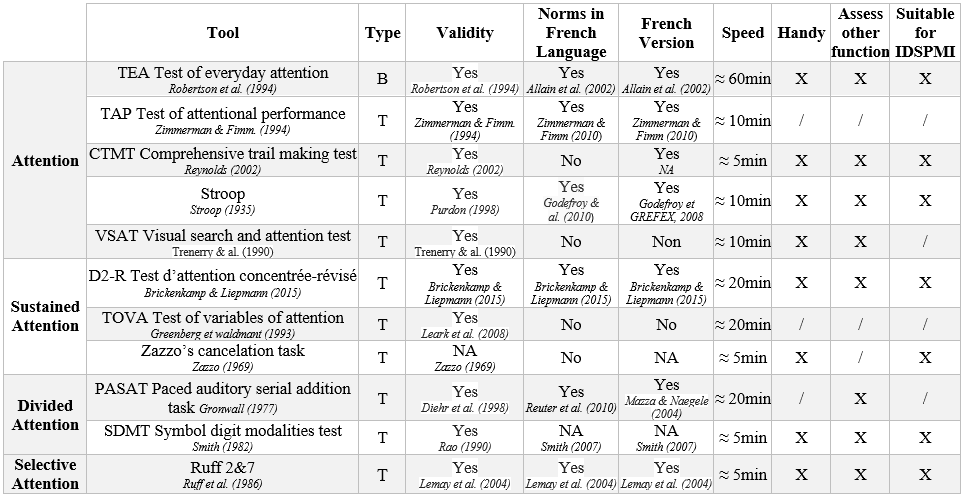
**

***Legend: NA:*** *Unofficial, not published or not relevant for this tool;* ***/*** *: Not relevant ;* ***B :*** *Battery ;* ***I :*** *Interview ;* ***Q :*** *Questionnaire ;* ***T :*** *Test.*

**Table 2: Principal tools useable and available in French language for processing speed assessment**

**
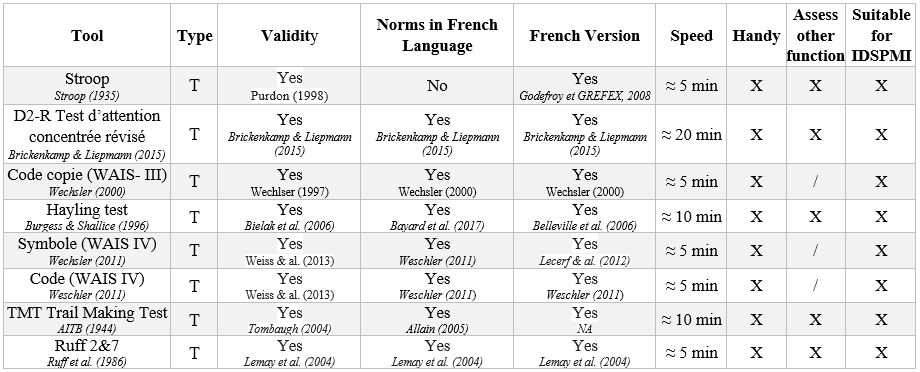
**

***Legend: NA:*** *Unofficial, not published or not relevant for this tool;* ***/*** *: Not relevant ;* ***B :*** *Battery ;* ***I :*** *Interview ;* ***Q :*** *Questionnaire ;* ***T :*** *Test.*

**Table 3: Principal tools useable and available in French language for memory assessment**

***
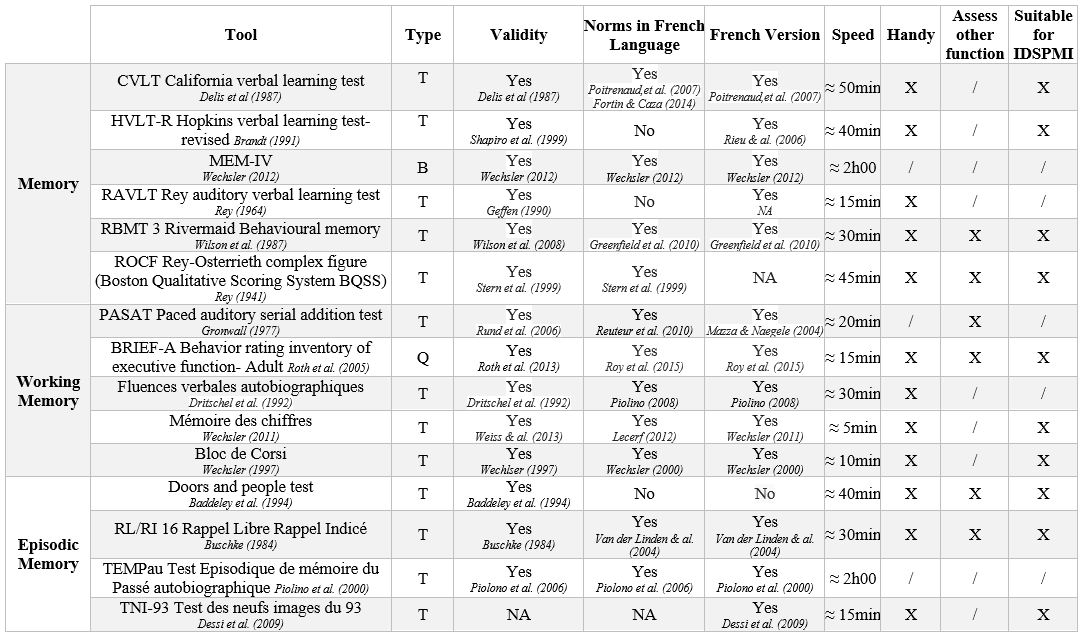
***

***Legend: NA:*** *Unofficial, not published or not relevant for this tool;* ***/*** *: Not relevant ;* ***B :*** *Battery ;* ***I :*** *Interview ;* ***Q :*** *Questionnaire ;* ***T :*** *Test.*

**Table 4: Principal tools useable and available in French language for executive functions assessment**

***
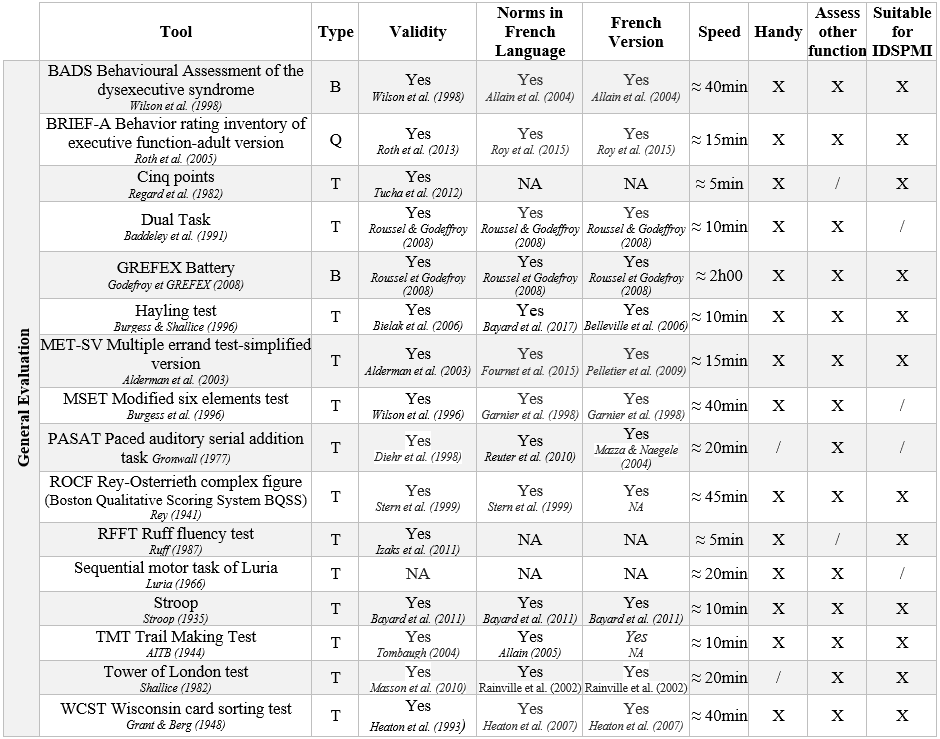
***

***Legend: NA:*** *Unofficial, not published or not relevant for this tool;* ***/*** *: Not relevant ;* ***B :*** *Battery ;* ***I :*** *Interview ;* ***Q :*** *Questionnaire ;* ***T :*** *Test.*

**
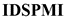
**
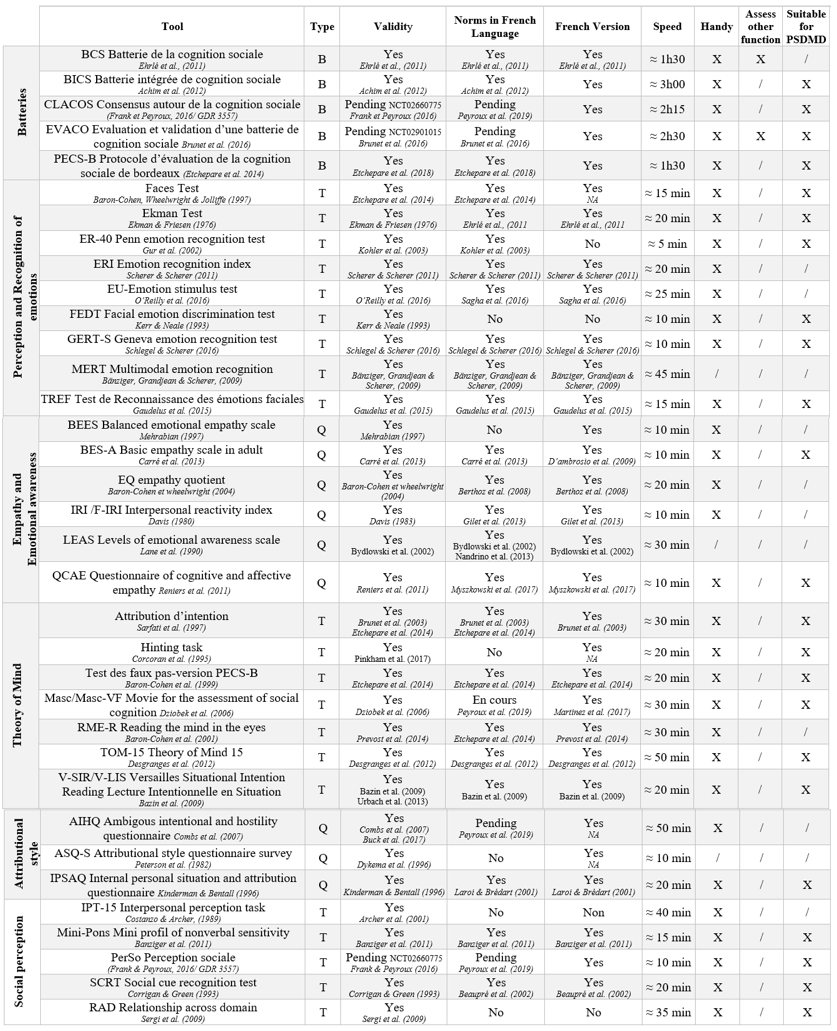
**T****able 5: Principal tools useable and available in French language for social cognition assessment**

***Legend: NA:*** *Unofficial, not published or not relevant for this tool;* ***/*** *: Not relevant ;* ***B :*** *Battery ;* ***I :*** *Interview ;* ***Q :*** *Questionnaire ;* ***T :*** *Test.*

**Table 6: Principal tools useable and available in French language for the assessment of metacognition**


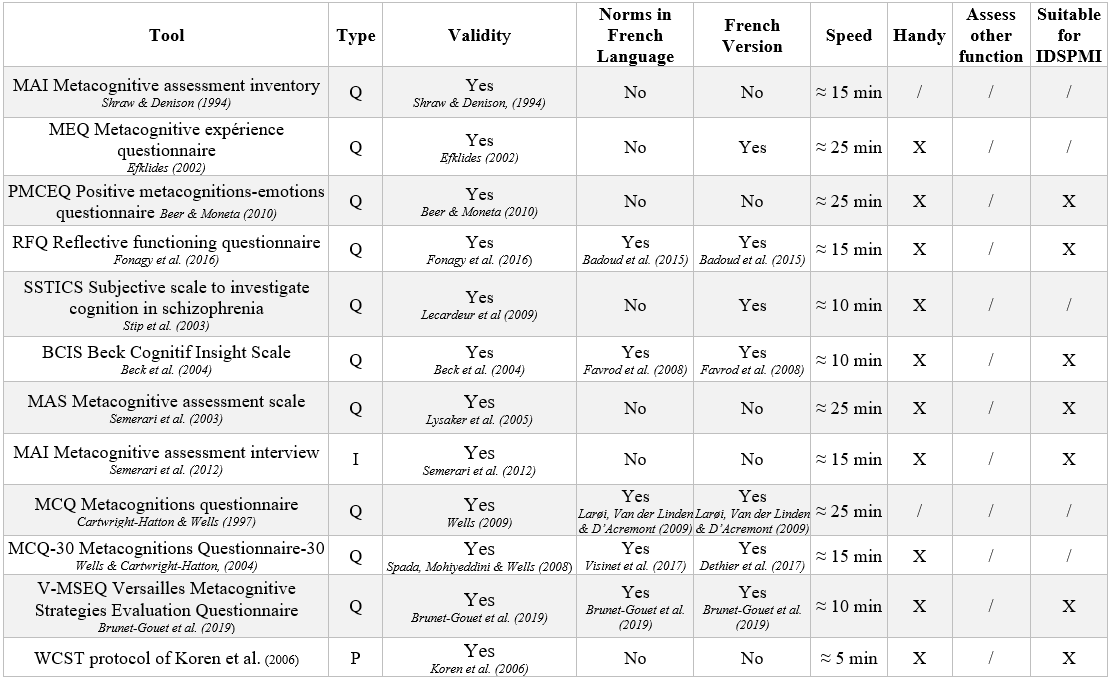


***Legend: NA:*** *Unofficial, not published or not relevant for this tool;* ***/*** *: Not relevant ;* ***B :*** *Battery ;* ***I :*** *Interview ;* ***Q :*** *Questionnaire ;* ***T :*** *Test.*

**Reference list**

Achim, A. M., Ouellet, R., Roy, M. A., & Jackson, P. L. (2012). Mentalizing in first-episode psychosis. *Psychiatry Research*, *196*(2-3), 207-213.

Alderman, N., Burgess, P.W., Knight, C. & Henman, C. (2003). Ecological validity of a simplified version of the multiple errands shopping test. *Journal of the International Neuropsychological Society*, *9*(1), 31-44.

Allain, P., Forgeau, M., Mohamed, Z.K., Etcharry-Bouyx, F. & Le Gall, D. (2002). Evaluation des troubles attentionnels chez des traumatisés crâniens sévères: intérêt d'une adaptation francophone du Test of Every day Attention. *Revue de neuropsychologie*, *12*(3), 401-435.

Allain, P., Roy, A., Kefi, Z., Pinon, K., Etcharry-Bouyx, F. & Le Gall, D. (2004). Fonctions exécutives et traumatisme cranien sévère: évaluation à l'aide de la "Behavioural Assessement of the Dysexecutive Syndrome". *Revue de neuropsychologie*, *14*(3), 285-324.

Allain, P., Roy, A., Pinon, K., Kefi, Z., Etcharry-Bouyx, F., Barre, J. ... & LE GALL, D. (2005). Rôle de l’âge sur les performances à quatre tests explorant le fonctionnement exécutif: Résultats obtenus auprès de 215 sujets sains. *L'Année gérontologique*, *19*(1), 78-89.

Archer, D., Costanzo, M. & Akert, R. (2001). The Interpersonal Perception Task (IPT): Alternative approaches to problems of theory and design. In J. A. Hall & F. J. Bernieri (Eds.), *Interpersonal sensitivity: Theory and measurement* (pp. 161–182). Mahwah, NJ: Erlbaum

Army Individual Test Battery. (1944). *Manual of Directions and Scoring*. Washington, DC : War Department, Adjutant General’s Office.

Baddeley, A. D., Bressi, S., Della Sala, S., Logie, R. & Spinnler, H. (1991). The decline of working memory in Alzheimer's disease: A longitudinal study. *Brain*, *114*(6), 2521-2542.

Baddeley, A., Emslie, H. & Nimmo-Smith, I. (1994*). The Doors and People Test: A test of visual and verbal recall and recognition*. Bury St-Edmunds, UK: Thames Valley Test Company.

Badoud, D., Luyten, P., Fonseca-Pedrero, E., Eliez, S., Fonagy, P. & Debbané, M. (2015). The French version of the Reflective Functioning Questionnaire: validity data for adolescents and adults and its association with non-suicidal self-injury. *PloS one*, *10*(12), e0145892.

Bänziger, T., Grandjean, D. & Scherer, K.R. (2009). Emotion recognition from expressions in face, voice, and body: the Multimodal Emotion Recognition Test (MERT). *Emotion*, *9*(5), 691.

Baron-Cohen, S., Wheelwright, S. & Jolliffe, A. T. (1997). Is there a" language of the eyes"? Evidence from normal adults, and adults with autism or Asperger syndrome. *Visual cognition*, *4*(3), 311-331.

Baron-Cohen, S., O'riordan, M., Stone, V., Jones, R. & Plaisted, K. (1999). Recognition of faux pas by normally developing children and children with Asperger syndrome or high-functioning autism. *Journal of autism and developmental disorders, 29*(5), 407-418.

Baron-Cohen, S. & Wheelwright, S. (2004). The empathy quotient: an investigation of adults with Asperger syndrome or high functioning autism, and normal sex differences. *Journal of autism and developmental disorders*, 34(2), 163-175.

Bayard, S., Erkes, J. & Moroni, C. (2011). Victoria Stroop Test: normative data in a sample group of older people and the study of their clinical applications in the assessment of inhibition in Alzheimer's disease. *Archives of Clinical Neuropsychology*, *26*(7), 653-661.

Bayard, S., Gély-Nargeot, M.C., Raffard, S., Guerdoux-Ninot, E., Kamara, E., Gros-Balthazard, F. ... & Collège des Psychologues Cliniciens spécialisés en Neuropsychologie du Languedoc Roussillon (CPCN-Languedoc Roussillon). (2017). French version of the Hayling Sentence Completion Test, Part I: Normative data and guidelines for error scoring. *Archives of Clinical Neuropsychology*, *32*(5), 585-591.

Bazin, N., Brunet-Gouet, E., Bourdet, C., Kayser, N., Falissard, B., Hardy-Baylé, M.C. & Passerieux, C. (2009). Quantitative assessment of attribution of intentions to others in schizophrenia using an ecological video-based task: a comparison with manic and depressed patients. *Psychiatry Research*, *167*(1-2), 28-35.

Beaupré, M.G., King, S., Bauer, I., Hess, U., Debruille, J.B. & Corrigan, P.W. (2002). Validation of french-and english-canadian versions of the Social Cue Recognition Test. *Canadian Journal of Psychiatry. Revue Canadienne de Psychiatrie,* 47(1), 81–85.

Beck, A.T., Baruch, E., Balter, J.M., Steer, R.A. & Warman, D.M. (2004). A new instrument for measuring insight: the Beck Cognitive Insight Scale. *Schizophrenia research*, *68*(2-3), 319-329.

Beer, N. & Moneta, G.B. (2010). Construct and concurrent validity of the positive metacognitions and positive meta-emotions questionnaire. *Personality and Individual Differences*, *49*(8), 977-982.

Belleville, S., Rouleau, N. & Van der Linden, M. (2006). Use of the Hayling task to measure inhibition of prepotent responses in normal aging and Alzheimer’s disease. *Brain and cognition*, *62*(2), 113-119.

Berthoz, S., Wessa, M., Kedia, G., Wicker, B. & Grèzes, J. (2008). Cross-cultural validation of the empathy quotient in a French-speaking sample. *The Canadian Journal of Psychiatry*, 53(7), 469-477.

Bielak, A.A., Mansueti, L., Strauss, E. & Dixon, R.A. (2006). Performance on the Hayling and Brixton tests in older adults: Norms and correlates. *Archives of Clinical Neuropsychology*, *21*(2), 141-149.

Brandt, J. (1991). The Hopkins Verbal Learning Test: Development of a new memory test with six equivalent forms. *The Clinical Neuropsychologist*, *5*(2), 125-142.

Brickenkamp, R., Schmidt-Atzert, L. & Liepmann, D. (2015). D*2-R: test d'attention concentré révisé: manuel*. Hogrefe.

Brunet, E., Sarfati, Y. & Hardy-Baylé, M.C. (2003). Reasoning about physical causality and other's intentions in schizophrenia*. Cognitive neuropsychiatry*, 8(2), 129-139.

Brunet, E. (2016). Evaluation and Validation of Social Cognition Battery to Characterize Schizophrenic Patients Functioning (EVACO). Identification NO. NCT02901015 [https://clinicaltrials.gov/ct2/show/NCT02901015](https://clinicaltrials.gov/ct2/show/NCT02901015?fbclid=IwAR38m8JRbyYbG5Re62OJSY83rvHr9BTK4ijY8qMYvvuOZdhgwUS3H5LAgOU)

Brunet-Gouet, E., Urbach, M., Ramos, V., Ehrminger, M., Aouizerate, B., Brunel, L. ... & Fond, G. (2019). Assessing metacognitive and help-seeking strategies in schizophrenia: design and psychometric validation of the Versailles Metacognitive Strategies Evaluation Questionnaire. *Clinical Rehabilitation*, 0269215519888784.

Buck, B., Iwanski, C., Healey, K.M., Green, M.F., Horan, W.P., Kern, R.S. ... & Penn, D. L. (2017). Improving measurement of attributional style in schizophrenia; A psychometric evaluation of the Ambiguous Intentions Hostility Questionnaire (AIHQ). *Journal of psychiatric research*, *89*, 48-54.

Burgess, P.W. & Shallice, T. (1996). Response suppression, initiation and strategy use following frontal lobe lesions. *Neuropsychologia*, *34*(4), 263-272.

Buschke, H. (1984). Cued recall in amnesia. *Journal of Clinical and Experimental Neuropsychology*, *6*(4), 433-440.

Bydlowski, S., Corcos, M., Paterniti, S., Guilbaud, O., Jeammet, P. & Consoli, S.M. (2002). Validation de la version française de l'échelle des niveaux de conscience émotionnelle [French validation study of the Levels of Emotional Awareness Scale]. *L'Encéphale: Revue de psychiatrie clinique biologique et thérapeutique, 28*(4), 310–320.

Cartwright-Hatton, S. & Wells, A. (1997). Beliefs about worry and intrusions: The Meta-Cognitions Questionnaire and its correlates. *Journal of anxiety disorders*, *11*(3), 279-296.

Carré, A., Stefaniak, N., D'ambrosio, F., Bensalah, L. & Besche-Richard, C. (2013). The Basic Empathy Scale in Adults (BES-A): Factor structure of a revised form. *Psychological assessment*, *25*(3), 679.

Combs, D.R., Penn, D.L., Wicher, M. & Waldheter, E. (2007). The Ambiguous Intentions Hostility Questionnaire (AIHQ): a new measure for evaluating hostile social-cognitive biases in paranoia. *Cognitive neuropsychiatry, 12*(2), 128-143

Corcoran, R., Mercer, G. & Frith, C.D. (1995). Schizophrenia, symptomatology and social inference: investigating “theory of mind” in people with schizophrenia. *Schizophrenia research*, 17(1), 5-13.

Corrigan, P.W. & Green, M.F. (1993). The Situational Feature Recognition Test: A measure of schema comprehension for schizophrenia. *International Journal of Methods in Psychiatric Research*, 3(1), 29–35.

Costanzo, M. & Archer, D. (1989). Interpreting the expressive behavior of others: The Interpersonal Perception Task. *Journal of Nonverbal Behaviour*, 13, 225-245.

D'Ambrosio, F., Olivier, M., Didon, D. & Besche, C. (2009). The basic empathy scale: A French validation of a measure of empathy in youth. *Personality and Individual Differences*, 46(2), 160–165.

Davis, M. H. (1980). *A multidimensional approach to individual differences in empathy.* JSAS. Austin: United-States.

Davis, M.H. (1983). Measuring individual differences in empathy: Evidence for a multidimensional approach. *Journal of Personality and Social Psychology, 44*(1), 113–126.

Desgranges, B., Laisney, M., Bon, L., Duval, C., Mondou, A., Bejanin, A. … & Muckle, G. (2012). TOM-15 : Une épreuve de fausses croyances pour évaluer la théorie de l'esprit cognitive. *Revue de neuropsychologie, 4*(3), 216-220.

Delis, D.C., Kramer, J.H., Kaplan, E. & Thompkins, B.A.O. (1987). *CVLT: California verbal learning test-adult version: manual*. Psychological corporation.

Dessi, F., Maillet, D., Metivet, E., Michault, A., Le Clésiau, H., Ergis, A.M. & Belin, C. (2009). Assessment of episodic memory in illitterate elderly. *Psychologie & NeuroPsychiatrie du vieillissement*, *7*(4), 287-296.

Dethier, V., Heeren, A., Bouvard, M., Baeyens, C. & Philippot, P. (2017). Embracing the Structure of Metacognitive Beliefs: Validation of the French Short Version of the Metacognitions Questionnaire. International *Journal of Cognitive Therapy*, 10(3), 219-233.

Diehr, M.C., Heaton, R.K., Miller, W. & Grant, I. (1998). The Paced Auditory Serial Addition Task (PASAT): norms for age, education, and ethnicity. *Assessment*, *5*(4), 375-387.

Dritschel, B.H., Williams, J.M.G., Baddeley, A.D. & Nimmo-Smith, I. (1992). Autobiographical fluency: A method for the study of personal memory. *Memory & cognition*, *20*(2), 133-140.

Dykema, J., Bergbower, K., Doctora, J.D. & Peterson, C. (1996). An attributional style questionnaire for general use. *Journal of Psychoeducational Assessment*, *14*(2), 100-108.

Dziobek, I., Fleck, S., Kalbe, E., Rogers, K., Hassenstab, J., Brand, M. ... & Convit, A. (2006). Introducing MASC: a movie for the assessment of social cognition. *Journal of autism and developmental disorders, 36*(5), 623-636.

Efklides A. (2002). Feelings as subjective evaluations of cognitive processing: How reliable are they? *Psychology: The Journal of the Hellenic Psychological Society, 9*, 163–184.

Ehrlé, N., Henry, A., Pesa, A. & Bakchine, S. (2011). Assessment of sociocognitive functions in neurological patients Presentation of a French adaptation of two tools and implementation in frontal dementia. *Geriatrie et psychologie neuropsychiatrie du vieillissement*, *9*(1), 117-128.

Ekman, P., & Friesen, W. V. (1976). Measuring facial movement. *Environmental psychology and nonverbal behavior*, *1*(1), 56-75.

Etchepare, A., Merceron, K., Amieva, H., Cady, F., Roux, S. & Prouteau, A. (2014). Évaluer la cognition sociale chez l'adulte: validation préliminaire du Protocole d'évaluation de la cognition sociale de Bordeaux (PECS-B). *Revue de neuropsychologie*, *6*(2), 138-149.

Etchepare, A., Roux, S., Destaillats, J.M., Cady, F., Fontanier, D., Couhet, G. & Prouteau, A. (2018). Éléments de validation du Protocole d’Évaluation de la Cognition Sociale de Bordeaux (PECS-B) en population générale et dans la schizophrénie. *Annales Médico-psychologiques, revue psychiatrique*. *In press.*

Favrod, J., Zimmermann, G., Raffard, S., Pomini, V. & Khazaal, Y. (2008). The Beck Cognitive Insight Scale in outpatients with psychotic disorders: further evidence from a French-speaking sample. *The Canadian Journal of Psychiatry*, *53*(11), 783-787.

Fonagy, P., Luyten, P., Moulton-Perkins, A., Lee, Y.W., Warren, F., Howard, S. ... & Lowyck, B. (2016). Development and validation of a self-report measure of mentalizing: The Reflective Functioning Questionnaire. *PLoS One, 11*(7), Article e0158678.

Fortin, A. & Caza, N. (2014). A validation study of memory and executive functions indexes in French-speaking healthy young and older adults. *Canadian Journal on Aging/La Revue canadienne du vieillissement*, *33*(1), 60-71.

Fournet, N., Demazières-Pelletier, Y., Favier, S., Lemoine, L. & Gros, C. (2015). Test des commissions révisé. In L. Hugonot-Diener, C. Thomas-Anthérion & F. Sellas (Eds), *GREMOIRE 2.* Louvain la Neuve : De Boeck Solal.

Frank, N. & Peyroux, E. (2016). Social Cognitive Assessment in Autism and Schizophrenia (ClaCoS). Identification NO. NCT02660775. Retrieved from [https://clinicaltrials.gov/ct2/show/NCT02660775](https://clinicaltrials.gov/ct2/show/NCT02660775?fbclid=IwAR3Lfmr8bkHutWOhO90XE2FAsr1iKubZ80Tn-74JVJFfJhnGZtRYYjNyqgg)

Garnier, C., Enot-Joyeux, F., Jokic, C., Le Thiec, F., Desgranges, B. & Eustache, F. (1998). Une évaluation des fonctions exécutives chez les traumatisés crâniens: L'adaptation du test des six éléments. *Revue de neuropsychologie*, *8*(3), 385-414.

Gaudelus, B., Virgile, J., Peyroux, E., Leleu, A., Baudouin, J. Y., & Franck, N. (2015). Mesure du déficit de reconnaissance des émotions faciales dans la schizophrénie. Étude préliminaire du test de reconnaissance des émotions faciales (TREF). *L'Encéphale*, *41*(3), 251-259.

Geffen, G., Moar, K.J., O'hanlon, A.P., Clark, C.R. & Geffen, L. B. (1990). Performance measures of 16–to 86-year-old males and females on the auditory verbal learning test. *Clinical Neuropsychologist*, *4*(1), 45-63.

Gilet, A.L., Mella, N., Studer, J., Grühn, D. & Labouvie-Vief, G. (2013). Assessing dispositional empathy in adults: A French validation of the Interpersonal Reactivity Index (IRI). *Canadian Journal of Behavioural Science/Revue canadienne des sciences du comportement*, 45(1), 42.

Godefroy, O. & GREFEX, O. (2008). *Fonctions exécutives et pathologies neurologiques et psychiatriques*. Évaluation en pratique clinique. Solal Marseille.

Godefroy, O., Azouvi, P., Robert, P., Roussel, M., LeGall, D. & Meulemans, T. (2010). Groupe de réflexion sur l’évaluation des fonctions exécutives. Dysexecutive syndrome: diagnostic criteria and validation study. *Annals of Neurology*, 68(6), 855-64.

Grant, D.A. & Berg, E. (1948). A behavioral analysis of degree of reinforcement and ease of shifting to new responses in a Weigl-type card-sorting problem. *Journal of experimental psychology*, *38*(4), 404-411.

Greenberg, L.M. & Waldmant, I. D. (1993). Developmental normative data on the Test of Variables of Attention (TOVA™). *Journal of Child Psychology and Psychiatry*, *34*(6), 1019-1030.

Greenfield, E., Wilson, B.A., Baddeley, A., Clare, L., Watson, P., Cockburn, J., Sopena, S., Tate, R., Crawford, J.R, Nannary, R. (2010). *Rivermead Behavioural Memory Test – troisième édition (RBMT 3)*. Paris: ECPA.

Gronwall, D.M.A. (1977). Paced auditory serial-addition task: a measure of recovery from concussion. *Perceptual and motor skills*, *44*(2), 367-373.

Gur, R.C., Sara, R., Hagendoorn, M., Marom, O., Hughett, P., Macy, L. ... & Gur, R.E. (2002). A method for obtaining 3-dimensional facial expressions and its standardization for use in neurocognitive studies. *Journal of neuroscience methods*, *115*(2), 137-143.

Heaton, R.K., Chelune, G.J., Talley, J.L., Kay, G.G, & Curtiss, G. (1993). Wisconsin Card Sorting Test (WSCT). Lutz, Florida : PAR Inc.

Heaton, R.K., Chelune, G.J., Talley, J.L., Kay, G.G, & Curtiss, G. (2007). Wisconsin Card Sorting Test (WSCT) French Version. Hoegrefe.

Izaks, G.J., Joosten, H., Koerts, J., Gansevoort, R.T. & Slaets, J.P. (2011). Reference data for the Ruff Figural Fluency Test stratified by age and educational level. *PLoS One*, *6*(2), e17045.

Kohler, C.G., Turner, T.H., Bilker, W.B., Brensinger, C.M., Siegel, S.J., Kanes, S.J. ... & Gur, R.C. (2003). Facial emotion recognition in schizophrenia: intensity effects and error pattern. *American Journal of Psychiatry*, *160*(10), 1768-1774.

Kerr, S.L. & Neale, J.M. (1993). Emotion perception in schizophrenia: Specific deficit or further evidence of generalized poor performance? *Journal of Abnormal Psychology*, 102(2), 312–318.

Kinderman P, Bentall RP. (1996) A new measure of causal locus: the internal, personal and situational attributions questionnaire. *Pers Individ Dif, 20,* 261-264.

Koren, D., Seidman, L.J., Goldsmith, M. & Harvey, P.D. (2006). Real-world cognitive—and metacognitive—dysfunction in schizophrenia: a new approach for measuring (and remediating) more “right stuff”. *Schizophrenia Bulletin*, *32*(2), 310-326.

Lane, R.D., Quinlan, D.M., Schwartz, G.E., Walker, P.A. & Zeitlin, S.B. (1990). The Levels of Emotional Awareness Scale: A cognitive-developmental measure of emotion*. Journal of personality assessment*, 55(1-2), 124-134.

Laroi, F. & Brédart, S. (2001). Presentation of a french version of the Internal, Personal and Situational Attributions Questionnaire*. European review of applied psychology*, 52, 133-141.

Laroi, F., Van der Linden, M. & d’Acremont, M. (2009). Validity and reliability of a French version of the metacognitions questionnaire in a nonclinical population. *Swiss Journal of Psychology*, *68*(3), 125-132.

Leark, R.A., Greenberg, L.M., Kindschi, C.L., Dupuy, T.R. & Hughes, S.J. (2008). TOVA professional manual. Los Alamitos, CA: TOVA Company.

Lecardeur, L., Briand, C., Prouteau, A., Lalonde, P., Nicole, L., Lesage, A. & Stip, E. (2009). Preserved awareness of their cognitive deficits in patients with schizophrenia: Convergent validity of the SSTICS. *Schizophrenia Research*, *107*(2-3), 303-306.

Lecerf, T., Golay, P. & Reverte, I. (2012). Scores composites CHC pour la WAIS-IV: normes francophones. *Pratiques psychologiques*, *18*(4), 401-412.

Lemay, S., Bédard, M.A., Rouleau, I. & Tremblay, P.L. (2004). Practice effect and test-retest reliability of attentional and executive tests in middle-aged to elderly subjects. *The Clinical Neuropsychologist*, *18*(2), 284-302.

Luria, A.R. (1966). *Higher cortical functions in man*. New York: Plenum.

Lysaker, P.H., Carcione, A., Dimaggio, G., Johannesen, J.K., Nicolo, G., Procacci, M. & Semerari, A. (2005). Metacognition amidst narratives of self and illness in schizophrenia: Associations withneurocognition, symptoms, insight and quality of life. *Acta Psychiatrica Scandinavica,*112(1),64–71.

Martinez, G., Alexandre, C., Mam-Lam-Fook, C., Bendjemaa, N., Gaillard, R., Garel, P. ... & Krebs, M.O. (2017). Phenotypic continuum between autism and schizophrenia: Evidence from the Movie for the Assessment of Social Cognition (MASC). *Schizophrenia research*, *185*, 161-166.

Masson, J. D., Dagnan, D. & Evans, J. (2010). Adaptation and validation of the Tower of London test of planning and problem solving in people with intellectual disabilities. *Journal of Intellectual Disability Research*, *54*(5), 457-467.

Mazza, S. & Naegele, B. (2004). *Test d'attention soutenue: PASAT modifié Adaptation française.*Collection: Tests et matériels en orthophonie : Solal.

Mehrabian, A. (1997). Relations among personality scales of aggression, violence, and empathy: Validational evidence bearing on the Risk of Eruptive Violence Scale. *Aggressive Behavior, 23*, 433-445.

Myszkowski, N., Brunet-Gouet, E., Roux, P., Robieux, L., Malézieux, A., Boujut, E. & Zenasni, F. (2017). Is the Questionnaire of Cognitive and Affective Empathy measuring two or five dimensions? Evidence in a French sample. *Psychiatry research*, *255*, 292-296.

Nandrino, J.L., Baracca, M., Antoine, P., Paget, V., Bydlowski, S. & Carton, S. (2013). Level of emotional awareness in the general French population: Effects of gender, age, and education level. International *Journal of Psychology*, 48(6), 1072-1079.

O’Reilly, H., Pigat, D., Fridenson, S., Berggren, S., Tal, S., Golan, O. ... & Lundqvist, D. (2016). The EU-emotion stimulus set: a validation study. *Behavior research methods*, *48*(2), 567-576.

Pelletier, Y., Lemoine, L., Gros, C. & Fournet, N. (2009). *Test des commissions modifié*. *GRECO*.

Peterson, C. & Villanova, P. (1988). An expanded attributional style questionnaire. *Journal of Abnormal Psychology*, *97*(1), 87.

Peyroux, E., Prost, Z., Danset-Alexandre, C., Brenugat-Herne, L., Carteau-Martin, I., Gaudelus, B. ... & Houy-Durand, E. (2019). From “under” to “over” social cognition in schizophrenia: Is there distinct profiles of impairments according to negative and positive symptoms? *Schizophrenia Research: Cognition*, *15*, 21-29.

Piolino, P., Desgranges, B., Eustache, F. & Eustache, F. (2000). *La mémoire autobiographique: théorie et pratique*. Marseille: Solal.

Piolino, P., Desgranges, B., Clarys, D., Guillery-Girard, B., Taconnat, L., Isingrini, M. & Eustache, F. (2006). Autobiographical memory, autonoetic consciousness, and self-perspective in aging. *Psychology and Aging, 21*(3), 510–525

Piolino, P. (2008). Evaluation et prise en charge des troubles de mémoire autobiographique en neuropsychologie. *Des amnésies psychogènes aux amnésies organiques*, 339-388.

Pinkham, A.E., Harvey, P.D. & Penn, D.L. (2017). Social Cognition Psychometric Evaluation: Results of the Final Validation Study. *Schizophrenia Bulletin*, 44(4), 737–748.

Poitrenaud, J., Deweer, B., Kalafat, M. & Van der Linden, M. (2007). *CVLT Test d’apprentissage et de mémoire verbale*. Paris: Editions du Centre de Psychologie Appliquée*.*

Prevost, M., Carrier, M.E., Chowne, G., Zelkowitz, P., Joseph, L. & Gold, I. (2014). The Reading the Mind in the Eyes test: validation of a French version and exploration of cultural variations in a multi-ethnic city. *Cognitive neuropsychiatry, 19*(3), 189-204.

Purdon, S.E. (1998). Olfactory identification and Stroop interference converge in schizophrenia. *Journal of Psychiatry and Neuroscience*, *23*(3), 163.

Rainville, C., Amieva, H., Lafont, S., Dartigues, J.F., Orgogozo, J.M. & Fabrigoule, C. (2002). Executive function deficits in patients with dementia of the Alzheimer's type A study with a Tower of London task. *Archives of Clinical Neuropsychology*, *17*(6), 513-530.

Rao, S.M. (1990). *A manual for the brief repeatable battery of neuropsychological tests in multiple sclerosis.* Milwaukee: Medical College of Wisconsin

Regard, M., Strauss, E. & Knapp, P. (1982). Children's production on verbal and non-verbal fluency tasks. *Perceptual and motor skills*, *55*(3), 839-844.

Reniers, R.L., Corcoran, R., Drake, R., Shryane, N.M. & Völlm, B.A. (2011). The QCAE: A questionnaire of cognitive and affective empathy. *Journal of personality assessment*, *93*(1), 84-95.

Reuter, F., Baumstarck-Barrau, K., Loundou, A., Pelletier, J. & Auquier, P. (2010). Paced auditory serial addition test: normative data in a French population. *Revue neurologique*, *166*(11), 944-947.

Rey, A. (1941). L'examen psychologique dans les cas d'encéphalopathie traumatique. (Les prob-lems.). [The psychological examination in cases of traumatic encepholopathy. Problems.]. Archives de Psychologie, 28, 215-285.

Rey, A. (1964). *L‘examen clinique en psychologie [Clinical tests in psychology]*. Paris: Presses Universitaires de France.

Reynolds, C. R. (2002). *Comprehensive trail making test (CTMT)*. Austin, TX: Pro-ED.

Rieu, D., Bachoud-Lévi, A.C., Laurent, A., Jurion, E. & Dalla Barba, G. (2006). Adaptation française du «Hopkins verbal learning test». *Revue neurologique*, *162*(6-7), 721-728.

Robertson, I.H., Ward, T., Ridgeway, V. & Nimmo-Smith, I. (1994). *The test of everyday attention (TEA).*Bury St. Edmunds, UK: Thames Valley Test Company.

Roth, R.M., Isquith, P. K. & Gioia, G.A. (2005). *Behavior Rating Inventory of Executive Function - Adult Version (BRIEF-A)*. Lutz, FL: Psychological Assessment Resources

Roth, R.M., Lance, C.E., Isquith, P.K., Fischer, A.S. & Giancola, P.R. (2013). Confirmatory factor analysis of the behavior rating inventory of executive function-adult version in healthy adults and application to attention-deficit/hyperactivity disorder. *Archives of clinical neuropsychology*, *28*(5), 425-434.

Roy, A., Besnard, J., Fournet, N., Lancelot, C., Le Gall, D. (2015). *BRIEF-A - Inventaire d’Evaluation Comportementale des Fonctions Executives - Version Adulte - Adaptation Française.* Hogrèfe France Editions, Paris

Roussel, M. & Godefroy, O. (2008). La batterie GREFEX: données normatives. In O. Godefroy & les membres du GREFEX (Eds.), *Fonctions exécutives et pathologies neurologiques et psychiatriques* (pp. 231-266). De Boeck Edition : Louvain la Neuve.

Ruff, R.M., Evans, R.W. & Light, R.H. (1986). Automatic detection vs controlled search: a paper-and-pencil approach. *Perceptual and motor skills*, *62*(2), 407-416.

Ruff, R.M., Light, R.H. & Evans, R.W. (1987). The Ruff Figural Fluency Test: a normative study with adults. *Developmental Neuropsychology*, *3*(1), 37-51.

Rund, B.R., Sundet, K., Asbjørnsen, A., Egeland, J., Landrø, N.I., Lund, A. ... & Hugdahl, K. (2006). Neuropsychological test profiles in schizophrenia and non‐psychotic depression. *Acta Psychiatrica Scandinavica*, *113*(4), 350-359.

Sagha, H., Matejka, P., Gavryukova, M., Povolný, F., Marchi, E. & Schuller, B.W. (2016). Enhancing Multilingual Recognition of Emotion in Speech by Language Identification. *Interspeech* 2949-2953.

Sarfati, Y., Hardy-Baylé, M.C., Besche, C. & Widlöcher, D. (1997). Attribution of intentions to others in people with schizophrenia: a non-verbal exploration with comic strips. *Schizophrenia research*, 25(3), 199-209.

Scherer, K. R. & Scherer, U. (2011). Assessing the ability to recognize facial and vocal expressions of emotion: Construction and validation of the Emotion Recognition Index. *Journal of Nonverbal Behavior*, *35*(4), 305.

Schlegel, K. & Scherer, K.R. (2016). Introducing a short version of the Geneva Emotion Recognition Test (GERT-S): Psychometric properties and construct validation. *Behavior research methods*, *48*(4), 1383-1392.

Schraw, G. & Dennison, R.S. (1994). Assessing metacognitive awareness. *Contemporary educational psychology*, *19*(4), 460-475.

Semerari, A., Carcione, A., Dimaggio, G., Falcone, M., Nicolò, G., Procacci, M. & Alleva, G. (2003). How to evaluate metacognitive functioning in psychotherapy? The metacognition assessment scale and its applications. *Clinical Psychology & Psychotherapy*, *10*(4), 238-261.

Semerari, A., Cucchi, M., Dimaggio, G., Cavadini, D., Carcione, A., Battelli, V. ... & Ronchi, P. (2012). The development of the Metacognition Assessment interview: instrument description, factor structure and reliability in a non-clinical sample. *Psychiatry research*, *200*(2-3), 890-895.

Sergi, M.J., Fiske, A.P., Horan, W.P., Kern, R.S., Kee, K.S., Subotnik, K.L. ... & Green, M.F. (2009). Development of a measure of relationship perception in schizophrenia. *Psychiatry research, 166*(1), 54-62.

Shallice, T. (1982). The Tower of London, specific impairments of planning. In D.E. Broadbent & L. Weiskrants (Eds.), *The neuropsychology of cognitive function* (pp. 199-209). London : The Royal Society.

Shapiro, A.M., Benedict, R.H., Schretlen, D. & Brandt, J. (1999). Construct and concurrent validity of the Hopkins Verbal Learning Test–revised. *The Clinical Neuropsychologist*, *13*(3), 348-358.

Smith, A. (1982). *Symbol digit modalities test*. Los Angeles, CA: Western Psychological Services.

Smith, A. (2007). *Symbol Digits Modalities Test: Manual.* Los Angeles Western Psychological Services.

Spada, M.M., Mohiyeddini, C. & Wells, A. (2008). Measuring metacognitions associated with emotional distress: Factor structure and predictive validity of the metacognitions questionnaire 30. *Personality and Individual differences*, *45*(3), 238-242.

Stern, R.A., Javorsky, D.J., Singer, E.A., Singer Harris, N.G.S., Somerville, J.A., Duke, L., Thompson, J. & Kaplan, E. (1999). "BQSS: The Boston Qualitative Scoring System for the Rey-Osterrieth Complex Figure: Professional manual." *Lutz, FL: Psychological Assessment Resources*.

Stip, E., Caron, J., Renaud, S., Pampoulova, T. & Lecomte, Y. (2003). Exploring cognitive complaints in schizophrenia: the subjective scale to investigate cognition in schizophrenia. *Comprehensive psychiatry*, *44*(4), 331-340.

Stroop, J.R. (1935). Studies of interference in serial verbal reactions. *Journal of experimental psychology*, *18*(6), 643.

Tombaugh, T.N. (2004). Trail Making Test A and B: normative data stratified by age and education. *Archives of clinical neuropsychology*, *19*(2), 203-214.

Tucha, L., Aschenbrenner, S., Koerts, J. & Lange, K. W. (2012). The Five-Point Test: Reliability, validity and normative data for children and adults. *PloS one*, *7*(9), e46080.

Trenerry, M.R., Crosson, B., DeBoe, J. & Leber, W.R. (1990). *Visual Search and Attention Test (VSAT)*. Psychological Assessment Resources.

Urbach, M., Brunet-Gouet, E., Bazin, N., Hardy-Baylé, M.C. & Passerieux, C. (2013). Correlations of theory of mind deficits with clinical patterns and quality of life in schizophrenia. *Frontiers in* psychiatry, 4, 30.

Van der Linden, M., Coyette, F., Poitrenaud, J., Kalafat, M., Calicis, F., Wyns, C. … Membres du GREMEM. (2004). L’épreuve de rappel libre/ rappel indicé à 16 items (RL/RI-16). In M. Van der Linden, S. Adam, A. Agniel, & Membres du GRENEM (Eds.), *L'évaluation des troubles de la mémoire : présentation de quatre tests de mémoire épisodique avec leur étalonnage*. (pp. 25-47). Marseille: Solal.

Visinet, A., Soumet-Leman, C., Baptista, A., Bungener, C. & Jouvent, R. (2017). Approche psychométrique de la métacognition: étude pilote en population clinique. *L'Encéphale*, *43*(2), 120-127.

Wechsler, D. (1997). *Wechsler Adult Intelligence Scale-Third Edition*. San Antonio, TX: The Psychological Corporation.

Wechsler, D. (2000). *WAIS-III: Echelle de l’intelligence de Wechsler pour adultes.* Paris : Éditions du Centre de Psychologie Appliquée.

Wechsler, D. (2011). *WAIS-IV: Echelle d'intelligence de Wechsler pour adultes*. Éditions du Centre de Psychologie Appliquée.

Wechsler, D. (2012). MEM-IV: échelle clinique de mémoire de Wechsler. ECPA.

Weiss, L.G., Keith, T.Z., Zhu, J. & Chen, H. (2013). WAIS-IV and clinical validation of the four-and five-factor interpretative approaches. *Journal of Psychoeducational Assessment*, *31*(2), 94-113.

Wells, A. & Cartwright-Hatton, S. (2004). A short form of the metacognitions questionnaire: properties of the MCQ-30. *Behaviour research and therapy*, *42*(4), 385-396.

Wells, A. (2009). *Metacognitive therapy for anxiety and depression*. Guilford press.

Wilson, B., Cockburn, J. & Halligan, P. (1987). Development of a behavioral test of visuospatial neglect. *Archives of physical medicine and rehabilitation*, *68*(2), 98-102.

Wilson, B. A., Alderman, N., Burgess, P. W., Emslie, H. & Evans, J. (1996). *Behavioural assessment of the dysexecutive syndrome*. Thames Valley Test Company.

Wilson B, Evans JJ, Emslie H et al. (1998) The development of an ecologically valid test for assessing patients with a dysexecutive syndrome*. Neuropsychological Rehabilitation,* 8(3), 213-28

Wilson, B.A., Greenfield, E., Clare, L., Baddeley, A., Cockburn, J., Watson, P., Tate, R., Sopen, S., Nannery, R. & Crawford, J.R. (2008) *The Rivermead Behavioral Memory Test – Third Edition*. London: Pearson Assessment

Zazzo, R. (1969). *Manuel pour l’examen psychologique de l’enfant II*. Neuchatel : Delachaux et Niestlé.

Zimmerman, P. & Fimm, B. (1994). *Tests d’évaluation de l’attention (TEA*). Psytest. Würselen, Deutschland.

Zimmerman P. & Fimm, B. (2010). *Tests d’évaluation de l’attention (TAP*). Version 2.2 Adaptation française de Michel Leclercq (Psytest). Herzogenrath.
